# Probiotics as an adjunctive therapy in periodontitis treatment—reality or illusion—a clinical perspective

**DOI:** 10.1038/s41522-024-00614-5

**Published:** 2024-12-16

**Authors:** Lamyae Baddouri, Matthias Hannig

**Affiliations:** 1 Clinic of Operative Dentistry, Periodontology and Preventive Dentistry, Saarland University, Homburg, Germany; 2 Pharmacy, Saarland University, Saarbrucken, Germany

**Keywords:** Clinical microbiology, Dentistry, Symbiosis

## Abstract

Periodontitis, a prevalent oral health issue, involves various microorganisms and clinical effects. This review examines probiotics as adjunctive therapy for periodontitis by analyzing forty clinical studies. Findings showed mixed results due to differences in study design, probiotic types, and clinical parameters; however, probiotics improved outcomes in severe cases. Caution is advised when interpreting these results, as longer follow-up periods reveal variability and potential regression in effects.

## Periodontitis

### Background

The complex oral microbiota constitutes a diverse community of microorganisms that inhabit the oral cavity and plays a crucial role in maintaining the balance and overall health of the oral environment^1^. These microorganisms form a symbiotic relationship with the immune system, modulate immune responses, and prevent pathogenic invasion. Perturbation and dysbiosis of the oral microbiota can lead to the development of caries and periodontitis, which are two common oral diseases^2^. Periodontitis is a chronic inflammatory disease that affects the supporting structure of the teeth, including the gums, periodontal ligament, and alveolar bone^3^.

Periodontitis can be categorized based on its severity and extent. The American Academy of Periodontology has defined a classification system that divides periodontitis into several stages based on severity, including stages of mild, moderate, and severe periodontitis^4^, and based on the etiology and diagnosis of aggressive periodontitis, we find Chronic periodontitis, and Aggressive periodontitis. However, there are cases which set in neither category. Therefore, they classify in Necrotizing periodontal disease, where periodontal disease is more complex due to factors such as undiagnosed syndrome, or due to variation that could be genetic, or immunological, or systemic^4^. Aggressive periodontitis can be categorized into a localized periodontitis or a generalized periodontitis^5^. Periodontitis could also be plaque induced or non-plaque-induced^6^.

Risk factors associated with the development of periodontitis diseases include poor oral hygiene habits, smoking^7^, diet^8^, hormonal changes^9^, a significant reduction of polymorphonuclear leukocytes^10^, genetic polymorphisms of genes involved in the production of cytokines^11^, as well as systemic conditions such as diabetes^12^, cardiovascular disease^13^, and immunosuppressant drugs or immunocompromised conditions^14^.

### Microorganisms associated with periodontal disease

The history of understanding the microorganisms associated with periodontal disease have come a long way. From believing that bacteria are simply secondary invaders rather than the primary cause of the disease^15^, to the identification of specific microorganisms that are commonly found in periodontal pockets and associated with the disease compared to health^16–19^. Following this, Loesche developed a ‘Specific plaque hypothesis’^20,21^. The key findings of this hypothesis are that periodontitis is a result of overgrowth of specific pathogens, characterized as the ‘red complex’ microorganisms, including *Porphyromonas gingivalis, Tannerella forsythia, and Treponema denticola*. These microorganisms have been strongly associated with the development and progression of periodontitis^22^. These microorganisms have been found to interact with the host immune system and manipulate inflammatory responses, leading to tissue destruction^23^. They produce a variety of virulence factors that contribute to their pathogenicity. These virulence factors include proteases, collagenases, lipases, and toxins, which facilitate tissue invasion, breakdown of connective tissues, and immune evasion^24^. *Actinobacillus actinomycetemcomitans* for instance, demonstrates its potent periodontopathogenicity by the ability to invade host cells and induce leukocytotoxicity^25^. For example, *Porphyromonas gingivalis* utilizes its fimbriae to adhere to oral surfaces and evade detection by the host immune cells^26^. Additionally, it produces lipopolysaccharides and outer membrane vesicles that can interfere with the host immune signaling pathways, promoting a chronic inflammatory state within the periodontal tissues^27^.

In addition to their direct effects on the host immune system and tissue destruction, the red complex microorganisms have also been implicated in the dysregulation of bone metabolism^28^, further contributing to the progression of periodontitis. These microorganisms have been shown to modulate osteoclast activity and interfere with the balance between bone resorption and formation, ultimately leading to alveolar bone loss and tooth mobility^29^, which are hallmark features of periodontitis. In addition to the red complex microorganisms, there are other microbial species that also contribute to the pathogenesis of the disease.

The orange complex microorganisms, classified as moderate pathogens including *Fusobacterium nucleatum*, *Prevotella intermedia*, and *Campylobacter* species have also been found to play a significant role in the progression of periodontitis by facilitating the colonization of other pathogens^30^, and creating an environment conducive to the growth and survival of pathogenic species^31^. In fact, these microorganisms appear to enhance the adherence of yellow, and purples complexes take place and facilitate the adhesion and survival of red complex microorganisms^32^.

Following the specific plaque hypothesis, an ‘Ecological catastrophe hypothesis’ was suggested by Marsh P.D.^33^, where he emphasizes the relationship between plaque bacteria and the host in health and disease, and implicated the concept of environmental factors influencing the selection and enrichment of pathogenic bacteria. A noteworthy remark that Marsh has pointed is that clinicians only treat symptoms of the disease rather than identifying the factor(s) driving the dysbiosis or so-called ‘ecological catastrophe’. Thus, modulating the shift in the oral microbiota back to sustainable homeostasis is the direction scientists in preventive dentistry should focus on. However, the polymicrobial synergy and dysbiosis of oral microbiota in periodontitis puts into question the red complex to something beyond and more complex^30^.

### Clinical changes associated with periodontal disease

Periodontitis is a result of untreated gingivitis associated to bacterial plaque accumulation, and alteration of the marginal gum, bleeding on probing, and an irreversible periodontal attachment loss with formation of pockets and recessions, and bone resorption with tooth mobility and exfoliation. Currently, there are no reliable clinical parameters to indicate existing periodontitis activity or to predict its occurrence, and existing clinical parameters vary in their degrees of accuracy and reliability^34^. However, the European Federation of Periodontology defined the clinical characteristics of periodontitis as the manifestation of three factors: Clinical Attachment Loss CAL, the presence of periodontal pockets, and bleeding on probing BOP, and gingival bleeding.

#### Pocket probing depths (PPD)

PPD refers to the depth measured in millimeters between the gingival margin and the base of the periodontal pocket, the progression of periodontal disease is related to an increase of PPD, which translates to deepening of the pockets due to inflammation and tissue destruction. PPD is monitored overtime to assess disease severity and response to treatment using a periodontal probe at various sites around each tooth. The value of mild to moderate pocket is >3 and <5 mm, moderate pockets have values between 5 mm and 7, and deep pockets have values of ≥7 mm^35^.

#### Bleeding on probing (BOP) percentage

BOP is the presence of bleeding from the gingival sulcus or periodontal pocket upon probing, an increase in BOP indicates the presence of periodontal disease, reflecting an active inflammation. BOP is assessed by gently probing the gingival sulcus or periodontal pockets, and the percentage of bleeding sites is recorded, this parameter helps to evaluate the oral hygiene practices and periodontal treatment. Presence or absence of BOP is used to determine the presence or absence of periodontitis. Studies have validated that non-bleeding gingival units may serve as an indication of periodontal stability^36,37^. Another study assessing BOP as a periodontal monitor revealed a very low predictive value for disease progression (6%), while the negative predictive value for absence was high (98%)^38^.

#### Clinical Attachment Level (CAL) loss

CAL loss is the amount of attachment loss between the tooth and surrounding tissues. CAL loss reflect the progression of the disease while a CAL gain reflects the regression of the disease. Thus, it is another crucial clinical parameter in the periodontal examination using a periodontal probe. The CAL value that indicates a progression of periodontal disease has varied in literature from a CAL loss of ≥2 mm^39^ to a loss of ≥3 mm^40^.

An analysis of periodontal disease progression vis-à-vis to CAL between healthy and diseased patients showed that subjects classified as “periodontally healthy” had an average of CAL of 1.1 mm, while “mild periodontitis” had a mean of 2.1 mm, and “severe periodontitis” had a mean of 2.5 mm^41^.

#### Plaque Index (PI)

Plaque Index is the amount of dental plaque present on tooth surfaces, an increased plaque accumulation reflects the initiation and progression of periodontal diseases, while a decrease of PI reflects the regression of the disease. Thus, it is also used to assess the response to treatment by visually assessing the presence and thickness of plaque on tooth surfaces. Currently, there are different methods to assess the plaque formation on the surface^42^. However, the development of several different types of index system is needed^43^.

### Limitations of conventional methods in periodontal therapy

Conventional periodontal treatment involves scaling and root planning, antibiotic treatment, and surgical procedures. The pathogenesis of periodontitis involves the colonization of pathogenic species, such as those in the orange complex, which produce virulence factors that contribute to tissue invasion and immune impairment. Additionally, the plaque hypothesis (discussed previously) has led to the exploration of targeted interventions, such as antibiotic treatment or non-surgical procedures, to eliminate the specific pathogen causing periodontal disease and promote healing. Scaling and root planning (SRP) is considered the gold standard in periodontitis therapy; it is a non-surgical therapy used to remove dental plaque and calculus by scaling and to smooth the infected root surfaces by root planning^44^. However, bacterial recolonization occurs shortly after treatment, and pathogenic microbiota is re-established within months after treatment^45^. Antibiotic treatment, in the other hand, is also used in treating periodontitis. However, it has become more complex due to the emergence of antibiotic-resistance bacteria^46^.

## Probiotics

### Background

Recently, there has been growing interest in the use of probiotics as a novel approach to prevent and treat periodontal disease. Probiotics are live microorganisms that, when administered in adequate amounts, confer health benefits to the host by modulating the composition and activity of the microbiota^47^ Probiotics have been studied as potential mechanisms for promoting oral health in periodontal disease, and caries^48^. Probiotic microorganisms, such as *Lactobacillus reuteri*, *Streptococcus salivarius*, and *Bifidobacterium dentium*, show promise in maintaining periodontal health by inhibiting the growth and virulence of pathogenic species in the oral microbiome, modulating the immune response, and promoting tissue healing processes^49^. The mechanisms in which probiotics act in the prevention and treatment of periodontal disease involve inhibiting the growth and virulence of pathogenic species by competing for resources^50^ and producing antimicrobial compounds^51^, modulating the immune response to control inflammation and promote healing^52^, and restoring microbial balance in the oral microbiome^53^. Inhibition of cariogenic microbial biofilm can also be achieved using probiotic microorganisms by competing with cariogenic bacteria for nutrients and adhesion sites^54^, producing antagonistic substances like lactic acid, hydrogen peroxide, and bacteriocins^55^.

During inflammation response, there is an overproduction of reactive oxygen species (ROS) causing an increase of oxidative stress and leading to tissue damage and disease progression^56^. Targeting oxidative stress and restoring its balanced level could lead to better management of apical periodontitis and enhance patients’ quality of life. Probiotics have been shown to activate antioxidant pathways and enhance the expression of various antioxidant enzymes and downregulate the inflammatory processes that lead to excessive ROS generation, thereby reducing oxidative damage, and decreasing inflammation in periodontal tissues^57^. Although there is not enough evidence in the use of probiotics in apical periodontitis, probiotics could offer an adjunctive benefit to apical periodontitis.

Recent research has highlighted the involvement of the NLRP3 inflammasome and NT-PRO-BNP in the progression of periodontitis. The NLRP3 inflammasome plays a central role in the activation of inflammatory responses, leading to the release of cytokines such as IL-1β, which exacerbates tissue destruction in periodontal disease^58,59^. Additionally, NT-PRO-BNP, commonly associated with cardiovascular health, has been linked to increased inflammation in periodontitis patients^60^.

Probiotics can promote anti-inflammatory pathways and antioxidant defenses, by inhibiting the damaging effects of NLRP3 inflammasome overactivation which can positively influence the host’s immune response by enhancing the production of anti-inflammatory cytokines and decreasing the production of pro-inflammatory cytokines such as IL-1β and TNF-α^61^, by inhibiting NLRP3 inflammasome activation.

Although the current review does not focus on the specific interactions between probiotics and these mediators, understanding their role in periodontitis pathophysiology provides a broader context for future studies. We suggest that ongoing research should further investigate how probiotic interventions may interact with these inflammatory pathways, as this could be a promising area for enhancing periodontitis treatment strategies^62^.

In oral health, probiotics have been found to improve clinical parameters such as pocket depth reduction, bleeding on probing reduction, and clinical attachment level gain^63^. Additionally, probiotics have been shown to improve the overall ecological balance of the oral microbiome, leading to a reduction in disease-associated dysbiosis^64^. One of the key strategies in the use of probiotics for periodontal disease is the modulation of the oral microbiome. This can be achieved by introducing beneficial probiotic microorganisms that could compete with pathogenic species for resources and adhesion sites, produce antimicrobial compounds, and promote a balanced microbial community. The available forms of probiotics for dental diseases include oral probiotic lozenges, gums, toothpaste, mouthwashes, and supplements^65^. Recent studies have shown promising results regarding the use of probiotics for the prevention and treatment of dental diseases^66–72^.

### The application vehicle

The choice of the delivery vehicle of probiotics in the oral cavity may influence the oral colonization and the cariogenic potential of probiotics^48^. The daily dose of probiotic intake may also influence the outcome of the intervention^73^. A range of vehicles was used in delivering probiotics as an adjunct to periodontal treatment, including lozenges, tablets, sachets, capsules, toothpaste, mouthwashes as well as local delivery using gel or drops based probiotics *(table)*. Table 1 mentions the basic characteristics of the studies like number of patients in both genders, age range, and inclusion criteria and exclusion or not of smokers and diabetics if reported.

**Table 1:** Basic characteristics of the studies

First author	Year	Number of patients	Age range	Gender M/F	Smokers (Y/N)	Diabetics (Y/N)	Inclusion criteria
Shimauchi et al.	2008	66	32–61	58/9	Y	NR	Periodontitis patients were recruited in this study. (Inclusion of smokers)
Vivekananda et al.	2010	30	35–50	19/11	N	N	Mild to moderate periodontal pockets (5–7 mm).
Teughels et al.	2013	30	≥ 35	15/15	N	N	Three previously untreated moderate to severe generalized adult periodontitis patients.
Vicario et al.	2013	20	NR	NR	N	N	Initial-to-moderate chronic periodontitis.
Szkaradkiewicz et al.	2014	38	31–46	18/20	N	NR	Moderate chronic periodontitis.
Tekce et al.	2015	40	35–50	18/22	N	N	at least two teeth with one approximal site each with a probing depth (PD) of 5–7 mm and gingival index (GI) of ≥2 in each quadrant.
İnce et al.	2015	30	30–50	17/13	N	N	At least two teeth with one approximal site each with a probing depth (PD) of 5–7 mm and gingival index (GI) of ≥2 in each quadrant.
Laleman et al.	2015	48	37–58	26/22	N	N	Untreated moderate to severe adult periodontitis.
Morales et al.	2016	28	35–68	14/14	Y	N	Previously untreated periodontitis.
Iwasaki et al.	2016	39	Average age: 67,6	13/23	NR	NR	Undergoing supportive periodontal therapy (SPT).
Penala et al.	2016	32	25–59	NR	N	N	Diagnosed with chronic periodontitis, and with clinically perceptible halitosis.
Chandra et al.	2016	30	25–50	NR	N	N	Two interdental suprabony pockets from the canine- premolar regions with 5–7 mm pocket depth in two different quadrants.
Mani et al.	2017	40	18–55	20/20	N	N	Mild to moderate chronic periodontitis.
Costacurta et al.	2018	40	18–70	20/20	NR	NR	Presence of at least two elements with PPD ≥ 4.
Invernici et al.	2018	41	≥30	NR	N	N	≥30% of sites with (PPD) ≥ 4 mm and CAL ≥ 4 mm and a minimum of five teeth with at least one site with CAL and PPD ≥ 5 mm.
Sajedinejad et al.	2018	50	24–52	45/45	N	N	Moderate to severe periodontitis.
Morales et al.	2018	47	≥35	NR	Y	N	Previously untreated for periodontitis. (Inclusion of smokers)
Alanzi et al.	2018	108 (101 completed the study)	13–15	101/0	Y	N	schoolboys
Boyeena et al.	2019	30	20–50	NR	N	N	At least 20 teeth, with periodontal pockets which bled on probing with probing depths (PDs) ≥ 5 mm
Paul et al.	2019	30	25–60	13/17	N	N	Mild to moderate periodontal pockets (5–7 mm).
Ikram et al.	2019	30	20–60	17/13	N	N	Mild to moderate periodontal pockets (5–7 mm).
Theodoro et al.	2019	34	30–56	15/13	Y	N	Smokers with periodontitis.
Pelekos et al.	2019	59	Average age: 53,3	15/26	N	N	Chronic periodontitis, that is with at least 2 non-adjacent teeth with PPD ≥ 5 mm.
Pelekos et al.	2020	40	Average age: 51,95	14/26	N	N	Stage III and IV periodontitis (presence of at least 2 probing sites in PPD ≥ 5 mm and radiographically determined bone loss)
Laleman et al.	2020	39	34–83	27/12	N	N	Previously non-surgically treated periodontitis, and with presence of ≥2 residual pockets.
Grusovin et al.	2020	20	31–70	8/12	Y	N	Treated GPIII-IVC otherwise healthy: patients were considered treated when they had no pus, BOP < 25%, PI < 25% and <9% of PPD > 6 mm
Vohra et al.	2020	127 (including 64 Shamma users)	Average age: 52.67	127/0	Y	N	Shamma users.
Butera et al.	2020	60	18–70	32/28	NR	NR	Presence of periodontal disease (stage II–III)
Bazyar et al.	2020	47	30–60	14/33	N	Y	Diabetes Mellitus and Chronic Periodontitis
Alshareef et al.	2020	40	25–58	NR	NR	N	diagnosed with chronic periodontitis.
Elsadek et al.	2020	60	35–75	26/17	N	Y	Patients diagnosed with stage III and grade C generalized periodontitis, and with type-2 diabetes mellitus.
Morales et al.	2021	47	≥35	26/21	Y	N	Patients with stage III periodontitis
Jebin et al.	2021	30	20–60	24/6	N	N	Stage II/Stage III and Grade A/Grade B periodontitis.
Pudgar et al.	2021	40	25–80	18/22	Y	N	Advanced periodontitis with a probing depth (PD) of ≥5 mm.
Ranjith et al.	2022	60	>30	33/27	N	N	Generalized stage II periodontitis, and Bleeding on probing in at least 30% sites.
Minić et al.	2022	80	35–55	NR	NR	N	Patients diagnosed with periodontitis.
Ramos et al.	2022	45	35–50	16/29	N	N	Stage II and III Grade B periodontitis.
Ghazal et al.	2023	60	≥35	NR	Y	N	Smokers with Stage III, Grade C generalized periodontitis.
Jardini et al.	2024	40	>30	NR	N	Y	T2DM diagnosed >5 years ago and Hb1Ac levels≥6.5 to≤9%, and diagnosed with periodontitis stage III and IV and grade B
Thierbach et al.	2024	28	≥18	NR	N	N	Stage III and IV periodontitis

N: No.

Y: Yes.

NR: Not reported.

### Aim of the review

This review evaluates the role of probiotics as an adjunctive therapy for periodontitis from a clinical perspective. We analyzed the baseline clinical parameters of each study, assessing how these metrics changed following intervention. Additionally, we outlined the inclusion and exclusion criteria for each study, detailing the selected probiotic strains, their administration methods, and frequency. This comprehensive overview aims to clarify the effects of probiotics in periodontal treatment.

The findings of this review hold significant clinical relevance by providing valuable insights into selecting appropriate probiotic strains, dosing, and intervention periods for future studies. Moreover, it emphasizes the importance for researchers working with periodontitis patients to ensure that the baseline clinical parameters of recruited individuals align with international periodontal guidelines. This alignment can enhance the validity and applicability of research outcomes, ultimately improving patient care.

## Clinical efficiency of adjunctive treatment probiotics in patients with chronic periodontitis, a controversy

### Different variables influence the outcome of the intervention

Table 2 mentions the probiotic strain, form and dosage used per day, the intervention period and the follow-up of the studies (Table 2), while Table 3 shows in the clinical parameters at baseline, after intervention, and -if exist- after follow-up (Table 3). These studies using probiotics as an adjunctive to periodontitis treatment showed a direct correlation between the period of intervention, the administrated dose with the gain of CAL and the reduction of PD. The follow up of treated patients also varies between studies, longer follow up shows regression with time in monitored clinical parameters and need of retreatment.

**Table 2:** Treatment type, frequency and duration

First Author	Year	Probiotic strain	Form	Dose CFU/day	Intervention Period (Weeks)	Follow up (Months)
Shimauchi et al.	2008	*L. salivarius WB21*	Tablets	2 * 10 ^ 9	8	NR
Vivekananda et al.	2010	*L. reuteri*	Lozenges	2 * 10 ^ 8	3	NR
Teughels et al.	2013	*L. reuteri*	Lozenges	2 * 10 ^ 8	12	NR
Vicario et al.	2013	*L. reuteri* (ATCC 55730 and ATCCPTA 5289)	Tablets	2 * 10 ^ 8	4	NR
Szkaradkiewicz et al.	2014	*L. reuteri* (ATCC PTA 5289)	Tablets	2 * 10 ^ 8	2	NR
Tekce et al.	2015	*L. reuteri*	Lozenges	2 * 10 ^ 8	3	6, 12
İnce et al.	2015	*L. reuteri*	Lozenges	2 * 10 ^ 8	3	6, 12
Laleman et al.	2015	*Streptococci*	Tablets	2 * ProBiora3	8	6
Morales et al.	2016	*L. rhamnosus*	Sachet	2 * 10 ^ 7	12	12
Iwasaki et al.	2016	Heat-killed *Lactobacillus plantarum L-137 (HK L-137)*	Capsules	2 * 10 ^ 7	12	3
Penala et al.	2016	*L. reuteri*, and *L.salivarius*	Capsules	8 * 10 ^ 9	2	3
Chandra et al.	2016	Probiotic (*Saccharomyces bouardi*)-Prebiotic (fructooligosaccharide, FOS) Mixture	Local delivery	NR	NR	3, 6
Mani et al.	2017	*Streptococcus salivarius* (DSM 13084) *Streptococcus salivarius* (DSM 14685) *Lactobacillus reuteri* (SD-5865) *Lactobacillus paracasei* (SD-5275)	Lozenges/Tablets	NR	4	2, 4
Costacurta et al.	2018	*L.reuteri* (ATCC-PTA 5289, and DSM 17938)	Tablets	10 ^ 8	4	NR
Invernici et al.	2018	*B.lactis HN019*	Lozenges	2 * 10 ^ 9	4	3
Sajedinejad et al.	2018	*Lactobacillus salivarius NK02*	Mouthwash	4 * 10 ^ 9	4	NR
Morales et al.	2018	*L. rhamnosus SP1*	Sachet	2 * 10 ^ 7	12	9
Alanzi et al.	2018	*Lactobacillus rhamnosus* GG (LGG) *and Bifidobacterium lactis* BB-12	Lozenges	4 * 10 ^ 8	4	NR
Boyeena et al.	2019	*L. acidophilus, L. rhamnosus, Bifidobacterium bifidus, Bifidobacterium longum*	Subgingivally delivered	1.8 * 10 ^ 9	12	NR
Paul et al.	2019	*L. brevis* CD2	Lozenges	2 * 10 ^ 9	3	3
Ikram et al.	2019	*L. reuteri* (ATCC 5289, and DSM17938)	Lozenges	4 * 10 ^ 8	3	1.5, 3
Theodoro et al.	2019	*L. reuteri*	Chewable tablets	2 * 10 ^ 8	3	3
Pelekos et al.	2019	*L. reuteri*	Lozenges	2 * 10 ^ 8	4	3, 6
Pelekos et al.	2020	*L. reuteri*	Lozenges	2 * 10 ^ 8	4	3, 6
Laleman et al.	2020	*L. reuteri*	Drops	4 * 10 ^ 8	12	6
Grusovin et al.	2020	*L. reuteri*	Lozenges	Reuterin OS ®, twice a day	12	6, 12
Vohra et al.	2020	*L. reutri* (ATCC-PTA 5289, and DSM 17938)	Lozenges	4 * 10 ^ 8	3	3, 6
Butera et al.	2020	Biorepair Peribioma	Toothpaste Biorepair® Peribioma^*TM*^ + chewingum Biorepair® Peribioma^*TM*^	Biorepair Peribioma	24	NR
Bazyar et al.	2020	*8 strains*	Multispecies probiotic supplement	NR	8	NR
Alshareef et al.	2020	*L. acidophilus, L. casei, Bifidobacterium bifidum, L. rhamnosus, and L. salivarius*.	Lozenges	NR	4	NR
Elsadek et al.	2020	*L. reuteri (*DSM-17938 and ATCC PTA5289)	Tablets	4 * 10 ^ 8	12	NR
Morales et al.	2021	*L.Rhamnosus SP1*	Sachet	2 * 10 ^ 7	12	12
Jebin et al.	2021	*L. reuteri UBLRu-87*	Tablets	5 * 10 ^ 8	4	3
Pudgar et al.	2021	*L. brevis and L. plantarum*	Gel (locally) + lozenges	NR	12	NR
Ranjith et al.	2022	*L. acidophilus, L. rhamnosus, B. longum and S. boulardii*.	Mouthwash	2.5 * 10 ^ 9	4	3
Minić et al.	2022	*L. acidophilus, Bifidobacterium infantis,Enterococcus faecium*	Local delivery	2 * 10 ^ 9 of *L.acidophilus*, 10 ^ 7 of *Bifidobacterium, and* 10 ^ 6 of *Enterococcus faecium*	5 days	1
Ramos et al.	2022	*L. reuteri (*DSM-17938 and ATCC PTA5289)	Lozenges	4 * 10 ^ 8	3	1 and 3.
Ghazal et al.	2023	*L. reuteri*	Tablets	4 * 10 ^ 8	4	3
Jardini et al.	2024	*L. reuteri (*DSM-17938 and ATCC PTA5289)	Lozenges	2 * 10 ^ 8	3	1, 3 and 6.
Thierbach et al.	2024	*L. reuteri (*DSM-17938 and ATCC PTA5289)	Lozenges	1 * 10 ^ 8	12	NR

Dose CFU/day: the sum of the total CFU per day taken by the participants.

NR : not reported.

**Table 3:** Probiotic efficacy on the clinical parameters, its clinical significance vs conclusions by the authors

First Author	Year	PPD/PD (mm)	BOP (%)	CAL (mm)	Clinical Significance	Conclusion by the author (s)
Shimauchi et al.	2008	**At baseline:** Control smoker Grp: 2.8 Control non-smoker Grp: 2.2 Probiotic smoker Grp: 2.9 Probiotic non-smoker Grp: 2.4 **After 1** **month:** Data was provided in figure. **After 2** **months:** Data was provided in figure.	**At baseline:** Control smoker Grp: 22.4 Control non-smoker Grp: 11.8 Probiotic smoker Grp: 21.8 Probiotic non-smoker Grp: 18.4 **After 1** **month:** Data was provided in figure. **After 2** **months:** Data was provided in figure.	NR	The PD was <3 mm, but in this case, the study is looking at subjects at a high risk of periosontal disease which are smokers.	Our results indicate that probiotics could be useful in the improvement / maintenance of oral health in subjects at a high risk of periodontal disease.
Vivekananda et al.	2010	**At baseline:** Control Grp: 5.26 Probiotic Grp: 5.08 **After intervention:** Data was provided in figure.	Data was provided in figure.	Data was provided in figure.	No comment is made	*L. reuteri* Prodentis probiotic can be recommended during non-surgical therapy and the maintenance phase of periodontal treatment.
Teughels et al.	2013	**PPD Overall** **At baseline:** Control Grp: 4.32 Probiotic Grp: 4.15 **After intervention:** Control Grp: 2.93 Probiotic Grp: 2.73 **PPD Moderate pockets** **At baseline:** Control Grp: 4.84 Probiotic Grp: 4.77 **After intervention:** Control Grp: 3.12 Probiotic Grp: 2.94 **PPD Deep Pockets** **At baseline:** Control Grp: 7.21 Probiotic Grp: 7.27 **After intervention:** Control Grp: 4.95 Probiotic Grp: 4.39	**At baseline:** Control Grp: 67.53 Probiotic Grp: 70.70 **After intervention:** Control Grp: 16.58 Probiotic Grp: 15.51	**CAL Overall** **At baseline:** Control Grp: 4.97 Probiotic Grp: 4.97 **After intervention:** Control Grp: 4.21 Probiotic Grp: 3.97 **CAL Moderate pockets** **At baseline:** Control Grp: 5.49 Probiotic Grp: 5.60 **After intervention:** Control Grp: 4.48 Probiotic Grp: 4.18 **CAL Deep Pockets** **At baseline:** Control Grp: 7.77 Probiotic Grp: 8.19 **After intervention:** Control Grp: 7.10 Probiotic Grp: 6.72	The difference of PD after intervention is <1 mm between the groups.	The results indicate that oral administration of *L. reuteri* lozenges could be a useful adjunct to SRP in chronic periodontitis.
Vicario et al.	2013	NR	**At baseline:** Control Grp: 40.0 Probiotic Grp: 55.3 **After intervention:** Control Grp: 47.0 Probiotic Grp: 29.3	NR	The PD and CAL values were not assessed in this study.	These data indicate that oral administration of *Lactobacillus reuteri* Prodentis improved the short-term clinical outcomes in non-smoking patients with initial-to-moderate chronic periodontitis.
Szkaradkiewicz et al.	2014	**At baseline:** Control Grp: 3.39 Probiotic Grp: 3.35 **After intervention:** Control Grp: 3.34 Probiotic Grp1a*: 3.06 Probiotic Grp1b*: 3.26	**SBI** **At baseline:** Control Grp: 1.73 Probiotic Grp: 1.69 **After intervention:** Control Grp: 1.75 Probiotic Grp1a*: 1.24 Probiotic Grp1b*: 1.67	**At baseline:** Control Grp: 3.49 Probiotic Grp: 3.47 **After intervention:** Control Grp: 3.56 Probiotic Grp1a*: 3.16 Probiotic Grp1b*: 3.53	The difference of PD after intervention is <1 mm between the groups.	Results obtained in this study indicate that application of oral treatment with tablets containing probiotic strain of *L. reuteri* induces in most patients with chronic periodontitis a significant reduction of pro-inflammatory cytokine response and improvement of clinical parameters (SBI, PPD, CAL).
Tekce et al.	2015	**At baseline:** Control Grp: 5.36 Probiotic Grp: 5.23 **After intervention:** Control Grp: 4.60 Probiotic Grp: 4.03 **After 6** **months:** Control Grp: 4.66 Probiotic Grp: 3.38 **After 12** **months:** Control Grp: 4.80 Probiotic Grp: 3.49	**At baseline:** Control Grp: 88.65 Probiotic Grp: 88.90 **After intervention:** Control Grp: 25.65 Probiotic Grp: 21.50 **After 6** **months:** Control Grp: 19.95 Probiotic Grp: 12.30 **After 12** **months:** Control Grp: 19.05 Probiotic Grp: 11.05	**At baseline:** Control Grp: – Probiotic Grp: – **After intervention:** Control Grp: – Probiotic Grp: – **After 6** **months:** Control Grp: 0.66 Probiotic Grp: 1.67 **After 12** **months:** Control Grp: 0.53 Probiotic Grp: 1.39	PD slightly increases after 12 months follow up	*L. reuteri-*containing lozenges may be a useful adjuvant agent to slow re-colonization and improve clinical outcomes of chronic periodontitis.
İnce et al.	2015	**PD** **At baseline:** Control Grp: 5.57 Probiotic Grp: 5.85 **After intervention:** Control Grp: 4.84 Probiotic Grp: 4.42 **After 6** **months:** Control Grp: 4.87 Probiotic Grp: 4.04 **After 12** **months:** Control Grp:5.01 Probiotic Grp: 4.15 **PD** ≥ **5** **mm (sites)** **At baseline:** Control Grp: 6.42 Probiotic Grp: 6.35 **After intervention:** Control Grp: 5.56 Probiotic Grp: 4.71 **After 6** **months:** Control Grp: 5.64 Probiotic Grp: 4.39 **After 12** **months:** Control Grp: 5.82 Probiotic Grp: 4.57	**At baseline:** Control Grp: 88.65 Probiotic Grp: 88.90 **After intervention:** Control Grp: 26.07 Probiotic Grp: 22.13 **After 6** **months:** Control Grp: 19.87 Probiotic Grp: 12.93 **After 12** **months:** Control Grp: 19.00 Probiotic Grp: 11.60	**At baseline:** Control Grp: – Probiotic Grp: – **After intervention:** Control Grp: – Probiotic Grp: – **After 6** **months:** Control Grp: 0.46 Probiotic Grp: 1.27 **After 12** **months:** Control Grp: 0.43 Probiotic Grp: 1.39	PD slightly increases after 12 months follow up	Lozenges containing *L. reuteri* may be a useful supplement in moderately deep pockets of patients with CP.
Laleman et al.	2015	**PPD Overall** **At baseline:** Control Grp: 4.59 Probiotic Grp: 4.50 **After intervention:** Control Grp: 3.26 Probiotic Grp: 3.15 **After 6** **months:** Control Grp: 2.98 Probiotic Grp: 2.99 **PPD** > **4** **mm** **At baseline:** Control Grp: 4.82 Probiotic Grp: 4.83 **After intervention:** Control Grp: 3.29 Probiotic Grp: 3.21 **After 6** **months:** Control Grp: 3.01 Probiotic Grp: 3.05 **PPD Deep Pockets (≥7** **mm)** **At baseline:** Control Grp: 7.18 Probiotic Grp: 7.13 **After intervention:** Control Grp: 4.78 Probiotic Grp: 4.76 **After 6** **months:** Control Grp: 3.76 Probiotic Grp: 3.93	**At baseline:** Control Grp: 85.55 Probiotic Grp: 87.44 **After intervention:** Control Grp: 28.31 Probiotic Grp: 27.74 **After 6** **months:** Control Grp: 30.11 Probiotic Grp: 26.98	PPD Overall CAL* **At baseline:** Control Grp: 5.36 Probiotic Grp: 5.22 **After intervention:** Control Grp: 4.66 Probiotic Grp: 4.47 **After 6** **months:** Control Grp: 4.60 Probiotic Grp: 4.51 **PPD** > **4** mm CAL* **At baseline:** Control Grp: 5.54 Probiotic Grp: 5.53 **After intervention:** Control Grp: 4.62 Probiotic Grp: 4.50 **After 6** **months:** Control Grp: 4.57 Probiotic Grp: 4.55 **PPD Deep Pockets (≥7** mm) CAL* **At baseline:** Control Grp: 8.26 Probiotic Grp: 8.09 **After intervention:** Control Grp: 6.87 Probiotic Grp: 6.77 **After 6** **months:** Control Grp: 6.44 Probiotic Grp: 6.44	The difference of PD after intervention is <1 mm between the groups, and no difference for CAL, and BOP.	No differences were detected when comparing the adjunctive use of a placebo or the investigated streptococci containing probiotic tablet after SRP.
Morales et al.	2016	**PD Overall** **At baseline:** Control Grp: 2.5 Probiotic Grp: 2.7 **After intervention:** Control Grp: 2.1 Probiotic Grp: 2.2 **After 6** **months:** Control Grp: 2.2 Probiotic Grp: 2.1 **After 12** **months:** Control Grp: 2.0 Probiotic Grp: 2.1 **PD Shallow sites** **At baseline:** Control Grp: 2.1 Probiotic Grp: 2.2 **After 12** **months:** Control Grp: 1.7 Probiotic Grp: 1.8 **Moderate Pockets** **At baseline:** Control Grp: 4.5 Probiotic Grp: 4.3 **After 12** **months:** Control Grp: 3.0 Probiotic Grp: 2.8 **PPD Deep Pockets (≥7** **mm)** **At baseline:** Control Grp: 7.9 Probiotic Grp: 7 **After 12** **months:** Control Grp: 4.7 Probiotic Grp: 3.7	**At baseline:** Control Grp: 33.8 Probiotic Grp: 41.1 **After intervention:** Control Grp: 23.6 Probiotic Grp: 28.2 **After 6** **months:** Control Grp: 27.9 Probiotic Grp: 29.7 **After 12** **months:** Control Grp: 25.4 Probiotic Grp: 29.3	**At baseline:** Control Grp: 4.9 Probiotic Grp: 4.2 **After intervention:** Control Grp: 4.2 Probiotic Grp: 3.8 **After 6** **months:** Control Grp: 4.3 Probiotic Grp: 3.9 **After 12** **months:** Control Grp: 4.8 Probiotic Grp: 4.1	The difference of PD after intervention is <1 mm between the groups in shallow and moderate sites, and significant improvement in probiotic groups in deep pockets.	The results of this trial indicate that oral administration *of L. rhamnosus* SP1 resulted in similar clinical improvements compared with SRP alone.
Iwasaki et al.	2016	Data was provided in mean (SD).	Data was provided in mean (SD).	Data was provided in mean (SD).	No comment is made.	These clinical findings suggest that daily HK L-137 intake can decrease the depth of periodontal pockets in patients undergoing supportive periodontal therapy.
Penala et al.	2016	**At baseline:** Control Grp: 3.19 Probiotic Grp: 3.12 **After 3** **months:** Data was provided in figure.	Data was provided in figure.	Data was provided in figure.	No comment is made.	The present investigation showed that the adjunctive use of probiotics offers clinical benefit in terms of pocket depth reduction in moderate pockets and reduced oral malodor parameters.
Chandra et al.	2016	**At baseline:** Control Grp: 5.52 Probiotic Grp: 5.66 **After 3** **months:** Control Grp:3.76 Probiotic Grp:3.19 **After 6** **months:** Control Grp:3.61 Probiotic Grp:2.19	**GI** **At baseline:** Control Grp: 2.09 Probiotic Grp: 2.19 **After 1** **week:** Control Grp:1.52 Probiotic Grp:1.19 **After 3** **months:** Control Grp:1.09 Probiotic Grp: 0.85 **After 6** **months:** Control Grp:1.80 Probiotic Grp: 0.90	**At baseline:** Control Grp: 3.52 Probiotic Grp: 3.57 **After 3** **months:** Control Grp:1.90 Probiotic Grp:1.42 **After 6** **months:** Control Grp:0.61 Probiotic Grp:0.58	Probiotic group showed better results at PD level; >1 mm difference.	The results suggest that *S. boulardii* is effective in improving the clinicalmeasures of periodontal disease. *S. boulardii* seems to thrive well in the subgingival environment and may function as an effective oral probiotic in subjects with periodontitis.
Mani et al.	2017	**PD** **At baseline:** Control Grp: 4.10 Probiotic Grp: 4.10 **After 2** **months:** Control Grp: 2.95 Probiotic Grp: 2.70 **After 4** **months:** Control Grp: 2.10 Probiotic Grp: 1.55	NR	**At baseline:** Control Grp: 4.20 Probiotic Grp: 4.15 **After 2** **months:** Control Grp: 2.95 Probiotic Grp: 2.80 **After 4** **months:** Control Grp: 2.35 Probiotic Grp: 1.85	The difference of PD after intervention is <1 mm between the groups, the BOP value was not assessed in this study.	Our results proved that daily oral supplementation of probiotics could be a useful adjunct to SRP in chronic periodontitis patients.
Costacurta et al.	2018	**At baseline:** Control Grp: 4.51 Probiotic Grp: 4.12 **After 1** **month:** Control Grp: 3.47 Probiotic Grp: 3.91	**At baseline:** Control Grp: 88.65 Probiotic Grp: 87.5 **After 1** **month:** Control Grp: 31.45 Probiotic Grp: 58.15	**At baseline:** Control Grp: 4.95 Probiotic Grp: 4.56 **After 1** **month:** Control Grp: 4.3 Probiotic Grp: 3.94	The difference of PD after intervention is <1 mm between the groups.	The subjects with CP, treated with SRP and probiotic, show some beneficial effect of *Lactobacillus reutri* with significant reduction of BOP and PPD.
Invernici et al.	2018	**PPD Overall** **At baseline:** Control Grp: 3.10 Probiotic Grp: 3.01 **After intervention:** Control Grp: 2.78 Probiotic Grp: 2.53 **After 3** **months:** Control Grp:2.85 Probiotic Grp: 2.49 **PPD** > **4** **mm** **At baseline:** Control Grp: 4.44 Probiotic Grp: 4.47 **After intervention:** Control Grp: 3.33 Probiotic Grp: 3.29 **After 3** **months:** Control Grp:3.50 Probiotic Grp: 3.19 **PPD Deep Pockets (≥7** **mm)** **At baseline:** Control Grp:7.26 Probiotic Grp: 7.27 **After intervention:** Control Grp:4.47 Probiotic Grp: 4.07 **After 3** **months:** Control Grp:4.64 Probiotic Grp: 3.75	**PPD Overall** **At baseline:** Control Grp: 35.00 Probiotic Grp: 30.80 **After intervention:** Control Grp: 24.05 Probiotic Grp: 17.05 **After 3** **months:** Control Grp: 30.71 Probiotic Grp: 18.80 **PPD** > **4** **mm** **At baseline:** Control Grp: 33.90 Probiotic Grp: 28.45 **After intervention:** Control Grp: 22.86 Probiotic Grp: 13.90 **After 3** **months:** Control Grp: 23.67 Probiotic Grp: 12.50 **PPD Deep Pockets (≥7** **mm)** **At baseline:** Control Grp: 6.00 Probiotic Grp: 5.60 **After intervention:** Control Grp: 1.90 Probiotic Grp: 1.25 **After 3** **months:** Control Grp: 2.90 Probiotic Grp: 1.25	PPD Overall CAL* **At baseline:** Control Grp: 3.42 Probiotic Grp: 3.26 **After intervention:** Control Grp: 3.13 Probiotic Grp: 2.77 **After 3** **months:** Control Grp: 3.24 Probiotic Grp: 2.77 **PPD** > **4** mm CAL* **At baseline:** Control Grp: 4.70 Probiotic Grp: 4.63 **After intervention:** Control Grp: 3.69 Probiotic Grp: 3.51 **After 3** **months:** Control Grp: 3.94 Probiotic Grp: 3.48 **PPD Deep Pockets (≥7** mm) CAL* **At baseline:** Control Grp: 7.62 Probiotic Grp: 7.48 **After intervention:** Control Grp: 5.08 Probiotic Grp: 4.36 **After 3** **months:** Control Grp: 5.55 Probiotic Grp: 4.03	The difference of PD after intervention is <1 mm between the groups.	The use of B. lactis HN019 as an adjunct to SRP promotes additional clinical, microbiological, and immunological benefits in the treatment of chronic periodontitis
Sajedinejad et al.	2018	**At baseline:** Control Grp: 2.55 Probiotic Grp: 2.67 **After intervention:** Data was provided in figure. **After 1** **month:** Data was provided in figure.	Data was provided in figure.	Data was provided in figure.	No comment is made.	Our findings suggest that probiotic mouthwash is healthy for daily use as an alternative for maintaining dental and periodontal health.
Morales et al.	2018	**At baseline:** Control Grp: 3.1 AB Grp: 2.9 Probiotic Grp: 2.7 **After 3** **months:** Control Grp: 2.4 AB Grp: 2.3 Probiotic Grp: 2.1 **After 9** **months:** Control Grp: 2.5 AB Grp: 2.3 Probiotic Grp: 2.2	**At baseline:** Control Grp: 52.5 AB Grp: 57.4 Probiotic Grp: 49.3 **After 3** **months:** Control Grp: 40.7 AB Grp: 43.6 Probiotic Grp: 39.2 **After 9** **months:** Control Grp: 45.9 AB Grp: 48.1 Probiotic Grp:42.4	**At baseline:** Control Grp: 4.7 AB Grp: 4.4 Probiotic Grp: 3.8 **After 3** **months:** Control Grp: 4.1 AB Grp: 4.0 Probiotic Grp: 3.4 **After 9** **months:** Control Grp: 4.3 AB Grp: 4.1 Probiotic Grp: 3.4	The PD value at a baseline is <3 mm and therefore not categorized as patients with periodontitis, and the difference of PD values after intervention is <1 mm between the groups.	All groups showed improvements in clinical and microbiological parameters at all time points evaluated.
Alanzi et al.	2018	NR	GI **At baseline:** Control Grp: 1.00 Probiotic Grp: 1.07 **After intervention:** Control Grp: 0.61 Probiotic Grp: 0.82	NR	Only Gingival index and plaque index were assessed in this study.	The short-term daily consumption of LGG and BB-12 probiotic lozenges improved the gingival health in adolescents and decreased the microbial counts of A. actinomycetemcomitans, and P. gingivalis.
Boyeena et al.	2019	**At baseline:** Probiotic Grp: 6.7 Probiotic +AB Grp: 7.0 AB Grp: 7.2 **After intervention:** Probiotic Grp: 3.7 Probiotic +AB Grp: 3.3 AB Grp: 5.6	SBI **At baseline:** Probiotic Grp: 3.7 Probiotic +AB Grp: 3.5 AB Grp: 3.1 **After intervention:** Probiotic Grp: 1.4 Probiotic +AB Grp: 1.0 AB Grp: 1.9	NR	The combination of antibiotics and probiotics showed better results compared to the groups.	Group A (SRP + P) and Group C (SRP + ATB + P) showed better results than Group B (SRP + ATB).
Paul et al.	2019	**At baseline:** Control Grp: 3.42 Probiotic Grp: 3.28 **After intervention:** Control Grp: 2.71 Probiotic Grp: 2.79 **After 3** **months:** Control Grp: 2.57 Probiotic Grp: 2.617	**BI score** **At baseline:** Control Grp: 0.90 Probiotic Grp: 1.73 **After intervention:** Control Grp: 0.51 Probiotic Grp: 0.62 **After 3** **months:** Control Grp: 0.71 Probiotic Grp: 0.54	**At baseline:** Control Grp: 3.58 Probiotic Grp: 3.58 **After intervention:** Control Grp: 2.83 Probiotic Grp: 3.12 **After 3** **months:** Control Grp: 2.77 Probiotic Grp: 3.13	The difference of PD after intervention is <1 mm between the groups.	The present study did not show a significant difference of using probiotic over SRP.
Ikram et al.	2019	**PPD** **At baseline:** AB Grp:4.77 Probiotic Grp:4.87 **After 1.5** **months:** AB Grp:3.64 Probiotic Grp:3.63 **After 3** **months:** AB Grp: 2.88 Probiotic Grp:2.91	**At baseline:** AB Grp: 0.79 Probiotic Grp: 0.79 **After 1.5** **months:** AB Grp: 0.74 Probiotic Grp: 0.74 **After 3** **months:** AB Grp: 0.67 Probiotic Grp: 0.68	**At baseline:** AB Grp: 4.03 Probiotic Grp: 3.78 **After 1.5** **months:** AB Grp: 3.66 Probiotic Grp: 3.40 **After 3** **months:** AB Grp: 3.31 Probiotic Grp: 3.04	The difference of PD after intervention is <1 mm between the groups.	The adjunctive use of *L. reuteri* and systemic antibiotics along with SRP showed similar improvement in all clinical periodontal parameters.
Theodoro et al.	2019	**At baseline:** Control Grp: 3.81 Probiotic Grp: 3.23 **After 3** **months**: Control Grp: 3.66 Probiotic Grp: 2.98	**At baseline:** Control Grp: 74.1 Probiotic Grp: 45.75 **After 3** **months**: Control Grp: 65.13 Probiotic Grp: 23.51	**At baseline:** Control Grp: 4.23 Probiotic Grp: 4.39 **After 3** **months**: Control Grp: 4.17 Probiotic Grp: 3.96	The PD value at a baseline is around 3 mm and therefore not categorized as patients with periodontitis, and the difference of PD values after intervention is <1 mm between the groups.	The adjuvant use of *L. reuteri* in the treatment of chronic periodontitis was effective in controlling gingival inflammation because reduced bleeding on probing which means reduced gingival inflammation and was effective in reducing deep pocket in manner clinically relevant.
Pelekos et al.	2019	**At baseline:** Control Grp: 3.1 Probiotic Grp: 3.5 **After 3** **months:** Control Grp: 2.7 Probiotic Grp: 3.0 **After 6** **months:** Control Grp: 2.6 Probiotic Grp: 2.9	**At baseline:** Control Grp: 69.1 Probiotic Grp: 59.5 **After 3** **months:** Control Grp: 37.4 Probiotic Grp: 42.2 **After 6** **months:** Control Grp: 29.6 Probiotic Grp: 2.9	**At baseline:** Control Grp: 4.9 Probiotic Grp: 4.2 **After 3** **months:** Control Grp: 4.0 Probiotic Grp: 4.6 **After 6** **months:** Control Grp: 4.0 Probiotic Grp: 4.6	The PD value at a baseline is slightly >3 mm, and the difference of PD after intervention is <1 mm between the groups.	The adjunctive use of probiotics with NSPT did not show any additional clinical effectiveness when compared to NSPT alone in the management of periodontitis
Pelekos et al.	2020	**At baseline:** Control Grp: 6.38 Probiotic Grp: 5.95 **After 3** **months:** Control Grp: 5.30 Probiotic Grp: 4.71 **After 6** **months:** Control Grp: 4.97 Probiotic Grp: 4.55	**At baseline:** Control Grp: 93.2 Probiotic Grp: 88.1 **After 3** **months:** Control Grp: 62.9 Probiotic Grp: 55.2 **After 6** **months:** Control Grp: 61.2 Probiotic Grp: 52.4	**At baseline:** Control Grp: 8.02 Probiotic Grp: 7.61 **After 3** **months:** Control Grp: 7.59 Probiotic Grp: 7.00 **After 6** **months:** Control Grp: 7.50 Probiotic Grp: 7.07	The difference of PD values after intervention is <1 mm between the groups.	A 28-day course of adjunctive probiotic *L. reuteri* lozenges improved CAL change at molar sites with ≥ 5 mm deep pockets and conferred a higher probability of shallow residual pocket depth. Presence of furcation-involvement and bleeding on probing worsened treatment outcomes.
Laleman et al.	2020	**PPD Overall** **At baseline:** Control Grp: 3.28 Probiotic Grp: 3.09 **After intervention:** Control Grp: 2.84 Probiotic Grp: 2.66 **After 6** **months:** Control Grp: 2.92 Probiotic Grp: 2.64 **PPD Moderate pockets (4** − **6** **mm)** **At baseline:** Control Grp: 4.68 Probiotic Grp: 4.56 **After intervention:** Control Grp: 3.55 Probiotic Grp: 3.36 **After 6** **months:** Control Grp: 3.67 Probiotic Grp: 3.35 **PPD Deep Pockets (≥7** **mm)** **At baseline:** Control Grp: 7.43 Probiotic Grp: 7.29 **After intervention:** Control Grp: 5.73 Probiotic Grp: 5.03 **After 6** **months:** Control Grp: 5.73 Probiotic Grp: 4.94	**At baseline:** Control Grp: 38 Probiotic Grp: 34 **After intervention:** Control Grp: 25 Probiotic Grp: 20 **After 6** **months:** Control Grp: 27 Probiotic Grp: 20	**CAL Overall** **At baseline:** Control Grp: 3.67 Probiotic Grp: 3.58 **After intervention:** Control Grp: 3.36 Probiotic Grp: 3.02 **After 6** **months:** Control Grp: 3.49 Probiotic Grp: 3.04 **CAL Moderate pockets (4** − **6** **mm)** **At baseline:** Control Grp: 5.01 Probiotic Grp: 5.04 **After intervention:** Control Grp: 4.05 Probiotic Grp: 3.66 **After 6** **months:** Control Grp: 4.21 Probiotic Grp: 3.73 **CAL Deep Pockets (≥7** **mm)** **At baseline:** Control Grp: 7.88 Probiotic Grp: 7.85 **After intervention:** Control Grp: 6.21 Probiotic Grp: 5.68 **After 6** **months:** Control Grp: 6.32 Probiotic Grp: 5.70	The difference of PD after intervention is <1 mm between the groups.	The adjunctive consumption of *L. reuteri* lozenges after re-instrumentation improved the PPD reduction, without an impact on pocket colonization with periodontopathogens.
Grusovin et al.	2020	**PPD Overall** **At baseline:** Control Grp: 2.23 Probiotic Grp: 2.23 **After intervention:** Control Grp: 2.15 Probiotic Grp: 2.05 **After 6** **months:** Control Grp: 2.15 Probiotic Grp: 1.96 **After 12** **months:** Control Grp: 1.92 Probiotic Grp: 1.76 **PPD** > **4** **mm** **At baseline:** Control Grp: 4.39 Probiotic Grp: 4.39 **After intervention:** Control Grp: 3.35 Probiotic Grp: 2.69 **After 6** **months:** Control Grp: 2.96 Probiotic Grp: 1.83 **After 12** **months:** Control Grp: 2.64 Probiotic Grp: 1.95	**At baseline:** Control Grp: 22.42 Probiotic Grp: 16.23 **After intervention:** Control Grp: 22.42 Probiotic Grp: 13.23 **After 6** **months:** Control Grp: 16.77 Probiotic Grp: 9.57 **After 12** **months:** Control Grp: 11.10 Probiotic Grp: 10.80	**PAL** **At baseline:** Control Grp: 3.24 Probiotic Grp: 3.24 **After intervention:** Control Grp: 3.05 Probiotic Grp: 3.06 **After 6** **months:** Control Grp: 3.05 Probiotic Grp: 2.95 **After 12** **months:** Control Grp: 2.74 Probiotic Grp: 2.74	The difference of PD after intervention is <1 mm between the groups, and no difference in BOP and PAL.	The use of *L. reuteri* probiotics lozenges improved some clinical outcomes in treated GPIII-IVC patients during maintenance therapy.
Vohra et al.	2020	**At baseline:** Control Grp: 6.5 Probiotic Grp: 6.2 **After 3** **months:** Control Grp: 5 Probiotic Grp: 4.5 **After 6** **months:** Control Grp: 5.6 Probiotic Grp: 5.6	**At baseline:** Control Grp: 71.2 Probiotic Grp: 66.4 **After 3** **months:** Control Grp: 50.5 Probiotic Grp: 40.1 **After 6** **months:** Control Grp: 58.4 Probiotic Grp: 60.5	**At baseline:** Control Grp: 4.1 Probiotic Grp: 4.6 **After 3** **months:** Control Grp: 4.2 Probiotic Grp: 4.4 **After 6** **months:** Control Grp: 4.2 Probiotic Grp: 4.5	The difference of PD after intervention is <1 mm between the groups.	Habitual shamma use compromises the outcome of SRP in patients with CP. Among patients that do not use any form of smokless tobacco product, SRP is an effective treatment modality for the treatment of CP, and this relationship is independent of use of adjunct PT.
Butera et al.	2020	**PPD** **At baseline:** Control Grp: 5.88 Toothpaste Grp: 5.67 Toothpaste + Chewing Gum Grp: 5.57 **After 3** **months:** Control Grp: 5.55 Toothpaste Grp: 4.67 Toothpaste+Chewing Gum Grp: 3.74 **After 6** **months:** Control Grp: 5.80 Toothpaste Grp: 4.46 Toothpaste + Chewing Gum Grp: 3.52	**At baseline:** Control Grp: 66.25 Toothpaste Grp:67.00 Toothpaste + Chewing Gum Grp: 66.15 **After 3** **months:** Control Grp: 61.25 Toothpaste Grp:39.00 Toothpaste+Chewing Gum Grp: 39.90 **After 6** **months:** Control Grp: 64.00 Toothpaste Grp:33.00 Toothpaste + Chewing Gum Grp: 21.50	**At baseline:** Control Grp: 5:83 Toothpaste Grp: 5.64 Toothpaste + Chewing Gum Grp: 5.36 **After 3** **months:** Control Grp: 5.66 Toothpaste Grp: 4.74 Toothpaste+Chewing Gum Grp: 3.76 **After 6** **months:** Control Grp: 5.57 Toothpaste Grp: 4.44 Toothpaste + Chewing Gum Grp: 3.46	The adjunct use of probiotics-based toothpaste combined with probiotic chewing gum improved the clinical parameter compared to the groups.	The relationship between the use of probiotics and improvement in clinical parameters is still unclear and deserves to be further explored.
Bazyar et al.	2020	**At baseline:** Control Grp: 4.50 Probiotic Grp: 4.30 **After intervention:** Control Grp: 4.04 Probiotic Grp: 3.47	**At baseline:** Control Grp: 24 Probiotic Grp: 23 **After intervention:** Control Grp: 22 Probiotic Grp: 19	**At baseline:** Control Grp: 3.08 Probiotic Grp: 3.26 **After intervention:** Control Grp: 2.95 Probiotic Grp: 2.73	Probiotics groups showed better clinical parameter results compared to control group.	It was observed that synbiotic supplementation with NSPT may be beneficial in improving inflammatory, antioxidant, and periodontal status in T2DM patients with CP.
Alshareef et al.	2020	**At baseline:** Control Grp: 2.61 Probiotic Grp: 2.55 **After 1** **month:** Control Grp:2.3 Probiotic Grp: 2.19	**BI** **At baseline:** Control Grp: 49.75 Probiotic Grp: 40.75 **After 1** **month:** Control Grp:40.82 Probiotic Grp: 32.15	**At baseline:** Control Grp: 3.49 Probiotic Grp: 3.57 **After 1** **month:** Control Grp: 3.14 Probiotic Grp: 3.14	The PD value at a baseline is <3 mm and therefore not categorized as patients with periodontitis, and the difference of PD values after intervention is <1 mm between the groups.	The probiotics might have a beneficial effect on clinical and immunological outcomes in the management of chronic periodontitis patients.
Elsadek et al.	2020	**PD** **At baseline:** Control Grp: 3.36 Probiotic Grp: 3.29 PDT Grp*: 3.14 **After 3** **month:** Control Grp: 2.74 Probiotic Grp: 2.81 PDT Grp*: 2.65	**BOP (0/1)** **At baseline:** Control Grp:0.69 Probiotic Grp: 0.83 PDT Grp*:0.74 **After 3** **month:** Control Grp:0.45 Probiotic Grp: 0.63 PDT Grp*: 0.51	**At baseline:** Control Grp:4.71 Probiotic Grp: 4.06 PDT Grp*: 4.35 **After 3** **month:** Control Grp:4.29 Probiotic Grp: 3.54 PDT Grp: 3.88	The difference of PD values after intervention is <1 mm between the groups.	PDT (photodynamic therapy) showed additional benefit in deep periodontal pockets and slightly modest reduction in HbA1c levels in DM patients. Further clinical trials are required with large sample size and longer follow up duration to ascertain the findings of the present clinical study.
Morales et al.	2021	**PPD (4–6** **mm)** **At baseline:** Control Grp: 4.6 Probiotic Grp: 4.5 AB Grp: 4.5 **After 3** **months:** Control Grp: 4.2 Probiotic Grp: 4.4 AB Grp: 4.4 **After 12** **months:** Control Grp: 4.2 Probiotic Grp: 4.3 AB Grp: 4.3 **PPD (**≥**7** **mm)** **At baseline:** Control Grp: 7.8 Probiotic Grp: 7.5 AB Grp: 7.5 **After 3** **months:** Control Grp: 7.6 Probiotic Grp: 7.4 AB Grp: 7.7 **After 12** **months:** Control Grp: 7.2 Probiotic Grp: 7.9 AB Grp: 7.1	NR	**PPD (4–6** **mm)** CAL* **At baseline:** Control Grp: 6.1 Probiotic Grp: 5.6 AB Grp: 5.9 **After 3** **months:** Control Grp: 6.0 Probiotic Grp: 6.0 AB Grp: 6.4 **After 12** **months:** Control Grp: 6.5 Probiotic Grp: 6.1 AB Grp: 6.4 **PPD (**≥**7** **mm)** CAL* **At baseline:** Control Grp: 9.5 Probiotic Grp: 8.4 AB Grp: 9.5 **After 3** **months:** Control Grp: 11.1 Probiotic Grp: 9.3 AB Grp: 10.4 **After 12** **months:** Control Grp: 10.6 Probiotic Grp: 7.9 AB Grp: 10.4	No additional benefits of probiotics compared to the other groups.	The use of probiotics or azithromycin as an adjunct to SRP failed to provide additional benefits in the treatment of stage III periodontitis. The benefits of these two treatment regimes as an adjunct to SRP remain unclear.
Jebin et al.	2021	**At baseline:** Control Grp: 5.20 Probiotic Grp: 5.27 **After intervention:** Control Grp: 4.80 Probiotic Grp: 4.31 **After 3** **months:** Control Grp: 4.35 Probiotic Grp: 3.6	**GI score** **At baseline:** Control Grp: 1.90 Probiotic Grp: 1.89 **After intervention:** Control Grp: 1.17 Probiotic Grp: 0.81 **After 3** **months:** Control Grp: 1.36 Probiotic Grp: 1.02	**At baseline:** Control Grp: 4.17 Probiotic Grp: 3.99 **After intervention:** Control Grp: 3.84 Probiotic Grp: 3.43 **After 3** **months:** Control Grp: 3.50 Probiotic Grp: 2.97	The difference of PD after intervention is <1 mm between the groups.	Probiotic chewable tablets containing *L. reuteri* may be a useful adjunct along with initial periodontal therapy to slow recolonization of periopathogens along with improvement in clinical outcomes of CP.
Pudgar et al.	2021	**At baseline:** Control Grp: 4.0 Probiotic Grp: 3.9 **After 3** **months:** Control Grp: 3.1 Probiotic Grp: 3.0	**At baseline:** Control Grp: 63.0 Probiotic Grp: 63.0 **After 3** **months:** Control Grp: 24.5 Probiotic Grp: 27.0	**At baseline:** Control Grp: 4.5 Probiotic Grp: 4.3 **After 3** **months:** Control Grp: 3.7 Probiotic Grp: 3.6	The difference of PD after intervention is <1 mm between the groups.	Patients with periodontitis benefit from adjunctive use of probiotics containing L. brevis and L. plantarum in terms of reduction of gingival bleeding. However, adjunctive probiotics increase the number of persisting diseased sites with PD > 4 mm and BOP.
Ranjith et al.	2022	**At baseline:** Control Grp: 3.0 Probiotic Grp: 3.4 **After 1** **month:** Control Grp: 2.5 Probiotic Grp: 2.67 **After 3** **months:** Control Grp: 2.74 Probiotic Grp: 2.65	NR	**At baseline:** Control Grp: 2.9 Probiotic Grp: 3.48 **After 1** **month:** Control Grp: 2.5 Probiotic Grp: 2.68 **After 3** **months:** Control Grp: 2.72 Probiotic Grp: 2.25	The PD value at a baseline is around 3 mm and therefore not categorized as patients with periodontitis, and the difference of PD after intervention is <1 mm between the groups.	The present study supports the use of probiotic mouthwash as an adjunct to mechanical therapy for the management of stage II periodontitis.
Minić et al.	2022	**At baseline:** Control Grp: 5.22 Probiotic Grp: 5.30 **After 7** **days**: Control Grp: 5.19 Probiotic Grp: 5.25 **After 1** **month:** Control Grp: 4.72 Probiotic Grp: 4.08	**At baseline:** Control Grp: 1.87 Probiotic Grp: 1.80 **After 7** **days**: Control Grp: 0.28 Probiotic Grp: 0.40 **After 1** **month:** Control Grp: 0.82 Probiotic Grp: 0.18	NR	The difference of PD values after intervention is <1 mm between the groups.	Based on the results of this pilot study, it can be said that, during periodontal treatment, topical application of probiotics in combination with SRP increases the effectiveness of conventional non-surgical therapy of periodontitis.
Ramos et al.	2022	**PD** **At baseline:** Control Grp: 3.76 AB Grp: 3.66 Probiotic Grp: 3.86 **After 1** **month:** Control Grp: 3.15 AB Grp: 2.91 Probiotic Grp: 3.27 **After 3** **months:** Control Grp: 3.03 AB Grp: 2.79 Probiotic Grp: 3.13	**At baseline:** Control Grp: 88.0 AB Grp: 93.0 Probiotic Grp: 88.9 **After 1** **month:** Control Grp: 49.8 AB Grp: 35.2 Probiotic Grp: 48.1 **After 3** **months:** Control Grp: 42.4 AB Grp: 28.0 Probiotic Grp: 40.3	**At baseline:** Control Grp: 4.13 AB Grp: 4.31 Probiotic Grp: 4.13 **After 1** **month:** Control Grp: 3.73 AB Grp: 3.73 Probiotic Grp: 3.58 **After 3** **months:** Control Grp: 3.50 AB Grp: 3.70 Probiotic Grp: 3.48	No additional benefits	After 3 months, none of the adjuvant therapies provided any additional benefit for subgingival instrumentation.
Ghazal et al.	2023	**PD** **At baseline:** AB Grp: 3.11 Probiotic Grp: 3.12 **After 1** **month:** AB Grp: 2.92 Probiotic Grp: 2.87 **After 3** **months:** AB Grp: 2.79 Probiotic Grp: 2.71	**At baseline:** AB Grp: 22.87 Probiotic Grp: 21.17 **After 1** **month:** AB Grp: 1.07 Probiotic Grp: 0.40 **After 3** **months:** AB Grp: 1.80 Probiotic Grp: 0.17	**At baseline:** AB Grp: 4.24 Probiotic Grp: 4.28 **After 1** **month:** AB Grp: 4.23 Probiotic Grp: 4.29 **After 3** **months:** AB Grp: 4.15 Probiotic Grp: 4.17	The difference of PD values after intervention is <1 mm between the groups.	Administration of probiotics and antibiotics along with NSPT yield statistically significant differences in PD and BOP from baseline to 3-month follow-up. However, between the group differences were not statistically significant for the periodontal parameters (AL, PD, and BOP).
Jardini et al.	2024	**At baseline:** Control Grp: 3.1 AB Grp: 2.9 Probiotic Grp: 2.7 **After 3** **months:** Control Grp: 2.4 AB Grp: 2.3 Probiotic Grp: 2.1 **After 6** **months:** Control Grp: 2.5 AB Grp: 2.3 Probiotic Grp: 2.2	**At baseline:** Control Grp: 52.5 AB Grp: 57.4 Probiotic Grp: 49.3 **After 3** **months:** Control Grp: 40.7 AB Grp: 43.6 Probiotic Grp: 39.2 **After 6** **months:** Control Grp: 45.9 AB Grp: 48.1 Probiotic Grp: 42.4	**At baseline:** Control Grp: 4.7 AB Grp: 4.4 Probiotic Grp: 3.8 **After 3** **months:** Control Grp: 4.1 AB Grp: 4.0 Probiotic Grp: 3.4 **After 6** **months:** Control Grp: 4.3 AB Grp: 4.1 Probiotic Grp: 3.4	The PD value at a baseline is <3 mm and therefore not categorized as patients with periodontitis, and the difference of PD values after intervention is <1 mm between the groups.	Subgingival instrumentation improved the clinical periodontal parameters in patients with T2DM. The use of L. reuteri probiotics had no additional effects compared with the placebo; however, there was a positive effect on the lipoprotein subfraction.
Thierbach et al.	2024	Data was provided in figure.	Data was provided in figure.	Data was provided in figure.	No comment is made.	The oral administration of one lozenge per day for 3 months with *L. reuteri* in supportive periodontal therapy might have a positive influence on clinical parameters in supportive periodontal therapy, depending on the individual.

*NR* Not Reported, *PAL*Probing Attachment Level, *SBI* Sulcular Bleeding Index, *GI* Gingival index, *AB Grp* Antibiotics Group, *PDT Grp* Photodynamic Therapy Group

CAL* measured at specific PD site.

Probiotic Grp1a* and Probiotic Grp1b*: Probiotic group in this study was divided into two sub-groups a and b depending on their results.

Thus, studies showing improvement of post probiotic intervention without evaluating the necessity of retreatment after an extended period, lack sufficient evidence to support the recommendation of probiotics as an adjunctive therapy in periodontitis. A study conducted by Vohra and colleagues utilized a probiotic-based lozenge containing *Lactobacillus reuteri* strains (ATCC-PTA 5289 and DSM 17938) as an adjunct to scaling and root planing (SRP). The findings revealed significant improvement in clinical parameters at the 3 month follow-up in comparison to the control group. However, by the 6 month follow-up, the probiotic test group demonstrated values closer to the placebo group, with differences of 0.9% in bleeding on probing (BOP), 0.1 mm in probing depth (PD), 0.3 mm in clinical attachment level (CAL), and plaque index (PI) values showing a significant difference at the 3 month mark but converging to the same difference value at the 6 month follow-up^74^. An additional noteworthy aspect, as detailed in the clinical parameters section, is that the evaluation of most of these parameters relies on visual assessment using a probe with a 1 mm margin of error. Consequently, and from a clinical perspective, relevance of significances related to <1 mm does not conclusively demonstrate the added benefits.

It is noteworthy that while probiotic therapy may demonstrate initial benefits post-intervention and sustained improvement for a few months thereafter, subsequent regression and the absence of additional benefits compared to control groups during long-term follow-up periods raise questions regarding the efficacy of this therapy.

Furthermore, it may be suggested that achieving a permanent shift in the oral microbiome towards a healthier state might necessitate long-term or even lifelong administration of probiotics. These observations highlight the importance of further research to elucidate the optimal duration and efficacy of probiotic therapy in promoting lasting oral health benefits.

In a study conducted by Yılmaz and colleagues, follow-up assessments were carried out on days 21, 90, 180, and 360. The findings demonstrated a notable improvement in clinical parameters until day 180. However, by day 360, all clinical parameter values experienced a slight increase, resulting in a difference of 1.18 mm in probing depth (PD), 0.65 in plaque index (PI), and 0.86 in clinical attachment level (CAL) gain^66^. A different work from the same group lead to the same observation^75^. In another study, a noteworthy improvement was noted, particularly in deep pockets, after a 3 month period. Importantly, no regression was observed in the test group throughout the entire follow-up period^67^. In a different study employing a lozenge based on Lactobacillus reuteri DSM 17938 and ATCC PTA 5389, improvements were observed for probing pocket depths (PPD) >4 mm. However, an intriguing increase was noted at the 9-month mark, similarly observed in the placebo group at the 12 month interval ^68^.

Baseline parameters hold significant importance in every study, particularly in research on periodontitis treatment. Emphasizing the probing depth (PD) baseline values are essential, as they serve as a key indicator of disease severity and are instrumental in evaluating the treatment response, as elucidated in the PD section. Assigning individuals without pre-existing periodontitis to categories such as chronic or aggressive periodontitis, and obtaining positive results, may raise questions about the appropriateness of the categorization. The American Academy of Periodontology^35^ and the European Federation of Periodontology^76^ share a value of >3 mm for a patient to be a periodontitis case.

Morales and colleagues assessed the effect of *Lactobacillus rhamnosus* in Non-Surgical Treatment of Chronic Periodontitis, using a baseline of 2.7 mm in test group and 2.5 in control group^77^. A separate work assessed the clinical effect of *Lactobacillus salivarius* NK02-based mouthwash, they have used a linear model to describe the baseline of clinical parameters, which gives a better visualization of the distribution of statistical values of clinical parameters, and the PD mean was around 2.6 and 2.7^78^. Same could be observed in other studies^79–83^. Providing a baseline value of each group rather than just the inter and intra group values, would give a better idea on the effect of probiotics in periodontitis treatment. Other studies have categorized Probing Depths into PD, and PD ≥ 5 mm^70^, or to moderate pockets and deep pockets PD ≥ 7 mm^71–73,84^. A recurrent observation in these studies is that probiotic interventions result in notable improvements, particularly in deep pockets, implying a potential role for probiotics as adjunctive therapy in individuals with aggressive periodontitis.

Clinical studies assessing the clinical and microbiological effects of probiotic based treatment differ not only in the baseline parameters, probiotic formulation, intervention period and frequency, and follow up, but also in their approach, most studies use an oral administration, meanwhile others took a different direction where they administrate probiotics locally^72,79,85–87^; one study assessed the effect of probiotics on different routes (locally and/or orally) as well as antibiotic treatment alone or with probiotics, better results were observed in combining both administrating routes. Interestingly, adjunctive probiotics increased the number of persisting diseased sites with PD > 4 mm and BOP%^71^. In a distinct study, a comparative analysis between probiotics alone and probiotics combined with scaling and root planing (SRP) was conducted, treating only two quadrants.

The probiotics+SRP group exhibited superior outcomes. However, after 42 days of leaving one quadrant untreated, the results mirrored those observed at day 21 in the SRP-treated quadrant. The coexistence of an untreated quadrant with a treated quadrant within the same oral cavity might have impacted the overall microbiome, potentially exerting a negative influence on the SRP-treated quadrant and providing a plausible explanation for the observed regression of results after 42 days^86^. Boyeena and colleagues conducted a study evaluating the effectiveness of probiotics compared to tetracycline fibers as adjunctive therapy to scaling and root planning (SRP). The combination of probiotics and antibiotics yielded the most favorable outcomes, with probiotics alone producing the second-best results. However, it is noteworthy that the local delivery of probiotics and antibiotic fibers necessitates refraining from brushing around the designated area, and the comfort of the patient does not appear to have been taken into consideration^71^. Recently, research directions are leaning toward symbiotic supplementation^84,85^, heat killed probiotics^88^, and photodynamic therapy in combination with probiotics^83^.

Table 4 summarize the clinical relevance related to adjunct periodontitis therapy, using a score scale of 0/+/++ of pre-mentioned work but excluding all papers with missing clinical parameters (PPD, BOP, and CAL) or provided in mean of total patients, in figures, as well as papers with a PPD value <3 mm at baseline. From a total of 26 research paper, 18 of them resulted a difference of <1 mm between probiotic groups and the other groups, 1 with no additional effect, and 7 with a positive effect (Table 4).

**Table 4:** Scoring of probiotic effect as an adjunctive therapy to periodontitis treatment from published studies

First author	Year	Title	Score 0/+/++
Teughels et al.	2013	Clinical and microbiological effects of *Lactobacillus reuteri* probiotics in the treatment of chronic periodontitis: a randomized placebo‐controlled study.	+
Tekce et al.	2015	Clinical and microbiological effects of probiotic lozenges in the treatment of chronic periodontitis: a 1‐year follow‐up study.	++
İnce et al.	2015	Clinical and microbiological effects of *Lactobacillus reuteri* probiotics in the treatment of chronic periodontitis: a randomized placebo‐controlled study.	+
Laleman et al.	2015	The effect of a streptococci containing probiotic in periodontal therapy: a randomized controlled trial.	+
Chandra et al.	2016	Effect of a locally delivered probiotic-prebiotic mixture as an adjunct to scaling and root planing in the management of chronic periodontitis.	++
Mani et al.	2017	Efficacy of oral probiotics as an adjunct to scaling and root planing in nonsurgical treatment outcome of generalized chronic periodontitis patients: A clinico-microbiological study.	+
Costacurta et al.	2018	Clinical effects of Lactobacillus reuteri probiotic in treatment of chronic periodontitis. A randomized, controlled trial.	+
Invernici et al.	2018	Effects of *Bifidobacterium* probiotic on the treatment of chronic periodontitis: A randomized clinical trial.	+
Boyeena et al.	2019	Comparison of efficacy of probiotics versus tetracycline fibers as adjuvants to scaling and root planing.	++
Paul et al.	2019	A double-blind, placebo-controlled study to assess the clinical and microbiological effects of a probiotic lozenge as an adjunctive therapy in the management of chronic periodontitis.	+
Pelekos et al.	2019	A double‐blind, paralleled‐arm, placebo‐controlled and randomized clinical trial of the effectiveness of probiotics as an adjunct in periodontal care.	+
Theodoro et al.	2019	Effects of *Lactobacillus reuteri* as an adjunct to the treatment of periodontitis in smokers: randomized clinical trial.	+
Laleman et al.	2020	A dual‐strain *Lactobacilli reuteri* probiotic improves the treatment of residual pockets: A randomized controlled clinical trial.	+
Grusovin et al.	2020	Clinical efficacy of Lactobacillus reuteri-containing lozenges in the supportive therapy of generalized periodontitis stage III and IV, grade C: 1 year results of a double-blind randomized placebo-controlled pilot study.	+
Vohra et al.	2020	Effectiveness of scaling and root planing with and without adjunct probiotic therapy in the treatment of chronic periodontitis among *shamma* users and non‐users: A randomized controlled trial.	+
Butera et al.	2020	Probiotic alternative to chlorhexidine in periodontal therapy: Evaluation of clinical and microbiological parameters.	++
Bazyar et al	2020	The impacts of synbiotic supplementation on periodontal indices and biomarkers of oxidative stress in type 2 diabetes mellitus patients with chronic periodontitis under non-surgical periodontal therapy. A double-blind, placebo-controlled trial.	++
Morales et al.	2021	Clinical effects of probiotic or azithromycin as an adjunct to scaling and root planning in the treatment of stage III periodontitis: a pilot randomized controlled clinical trial.	++
Jebin et al.	2021	Clinical effects of probiotic or azithromycin as an adjunct to scaling and root planning in the treatment of stage III periodontitis: a pilot randomized controlled clinical trial.	++
Ranjith et al.	2022	Probiotic mouthwash as an adjunct to mechanical therapy in the treatment of stage II periodontitis: A randomized controlled clinical trial.	+
Minić et al.	2022	Effect of the local probiotics in the therapy of periodontitis: A randomized prospective study.	+
Ramos et al.	2022	Effect of systemic antibiotic and probiotic therapies as adjuvant treatments of subgingival instrumentation for periodontitis: A randomized controlled clinical study.	0
Ghazal et al.	2023	A placebo‐controlled randomized clinical trial of antibiotics versus probiotics as an adjuvant to nonsurgical periodontal treatment among smokers with Stage III, Grade C generalized periodontitis.	+

Scoring key:

0= No effect observed between the tested groups.

+ = Comparison of PPD reduction after the intervention between the tested groups is <1 mm difference.

+ + = Positive effect, a PPD reduction after the intervention in probiotic group is over 1 mm compared to the other groups.

## Conclusion and future perspectives

Previously published systematic reviews provided conflicting results when examining the clinical efficacy of probiotics on periodontitis. In another hand, a systematic review and meta-analysis by Gheisary et al.^89^ have suggested that probiotic supplementation improves clinical parameters and reduces the periodontopathogens and pro-inflammatory markers in patients with periodontitis. Another systematic review by Ausenda et al.^90^ has presented the adjunctive probiotic effect over time and delivery method used which was more insightful compared to other systematic studies. A recent meta-analysis by Li et al.^91^ suggested that probiotic as an adjunctive therapy to scaling and root planning can improve the clinical outcome of chronic periodontitis patients and reduce periodontitis pathogens level. Interestingly, the European Association of Periodontology do not recommend the use of probiotics as an adjunct to subgingival instrumentation^92^.

The strength of this review relies on presenting all 40 clinical and experimental studies using probiotics as an adjunctive therapy to non-surgical periodontal therapy, and presenting clinical parameter changes over time, follow-up period and dose of probiotic were calculated in each study and presented by Colony-forming unit per day (CFU/ day) which provides important information on which strain, form, dose, duration might be most clinically useful. Based on the clinical outcome of studies that included a baseline of probing depth >3 mm, we can suggest that probiotics can somewhat improve the clinical outcome in few different cases, as single strain like *L*. *reuteri* like done by Tekce et al.^66^ with a dosage of 2 * 10^8 per day for a period of 3 weeks or in the form of sachet (Morales et al. 2021 and Jebin et al. 2021) with a lower dosage (2 * 10^7) but prolonged intervention (12 weeks)^93,94^. Probiotics also showed a positive outcome when used as a combination of strains such as the study of Boyeena et al.^71^. and and Bayzaar et al.^84^. Local delivery by the dentists could also be an option like the study of Chandra et al. where they used a combination of probiotic and prebiotics as an adjunctive therapy for periodontitis^85^.

The limitation of this review is the high heterogeneity in the studies. The different strains, forms, intervention period and administration route, in addition to the baseline parameters represent a limit. Another limitation of this study is that it focused only on the clinical parameters of the studies in details and have not included microbiological and immunological effects of probiotics as an adjunctive therapy in periodontitis as not all the studies have assessed these aspects.

Probiotic strain alone or combinations of strains, their form, dose, the intervention time, follow-up period, and inclusion criteria especially the baseline of the PPD in the clinical parameters are all factors that influence the results of the probiotic therapy.

Studies in probiotics as an adjunctive therapy showed a promise outcome in both clinical and microbiological parameters. However, addressing the challenges associated with probiotic interventions in periodontitis necessitates a multidimensional approach. In addition to evaluating clinical parameters, future research should emphasize the assessment of the potential side effects or safety concerns associated with probiotic administration. Long-term follow-up studies are crucial for elucidating the sustained effects of probiotics on periodontal health and the prevention of disease recurrence.

Moving forward, comprehensive clinical trials that consider the diverse characteristics of patients with chronic periodontitis, standardize probiotic interventions, and incorporate advanced microbiome analysis techniques will be instrumental in establishing the potential of probiotics as a viable adjunctive therapy in periodontal treatment. Thus, developing a personalized probiotic-based treatment is critical direction for future research to achieve a safer, more effective, and long-lasting results in preventive dentistry in general and periodontitis treatment specifically.

## References

[1] Wang, J. The human microbiome and personalized medicine. In, *Genomic and Personalized Medicine* (eds. Ginsburg, G. S., & Willard, H. F.) 166–172 (Elsevier, 2013).

[2] Montevecchi, M., Valeriani, L., Gatto, M. R., D’Alessandro, G. & Piana, G. Subgingival pathogens in chronic periodontitis patients affected by type 2 diabetes mellitus: a retrospective case-control study. *J. Periodontal. Implant Sci.* **51**, 409 (2021). 34965620 10.5051/jpis.2100180009 PMC8718332

[3] Wu, T., Shi, W., Loewy, Z. & He, X. Managing denture biofilm related diseases. *Dent. Open J.* **2**, 80–86 (2015).

[4] Mittal, V. et al. A practicable approach for periodontal classification. *Dent. Res J.* **10**, 697–703 (2013). PMC3872618 24379855

[5] Parameter on Aggressive Periodontitis. American academy of periodontology. *J. Periodontol.* **71**, 867–869 (2000). 10.1902/jop.2000.71.5-S.867 10875695

[6] Holmstrup, P. Non‐plaque‐induced gingival lesions. *Ann. Periodontol.* **4**, 20–29 (1999). 10863372 10.1902/annals.1999.4.1.20

[7] Kinane, D. F. & Chestnutt, I. G. Smoking and periodontal disease. *Crit. Rev. Oral. Biol. Med.* **11**, 356–365 (2000). 11021635 10.1177/10454411000110030501

[8] Casarin, M., da Silveira, T. M., Bezerra, B., Pirih, F. Q. & Pola, N. M. Association between different dietary patterns and eating disorders and periodontal diseases. *Front. Oral. Health* **4**, 1152031 (2023). 37035252 10.3389/froh.2023.1152031 PMC10075359

[9] Boyapati, R., Cherukuri, S., Bodduru, R. & Kiranmaye, A. Influence of female sex hormones in different stages of women on periodontium. *J. Midlife Health* **12**, 263 (2021). 35264831 10.4103/jmh.jmh_142_21 PMC8849144

[10] Johnson, N. W. et al. Detection of high‐risk groups and individuals for periodontal diseases. *J. Clin. Periodontol.* **15**, 276–282 (1988). 3292592 10.1111/j.1600-051x.1988.tb01584.x

[11] Shapira, L., Wilensky, A. & Kinane, D. F. Effect of genetic variability on the inflammatory response to periodontal infection. *J. Clin. Periodontol.* **32**, 72–86 (2005). 16128831 10.1111/j.1600-051X.2005.00810.x

[12] Grossi, S. G. & Genco, R. J. Periodontal disease and diabetes mellitus: a two‐way relationship. *Ann. Periodontol.* **3**, 51–61 (1998). 9722690 10.1902/annals.1998.3.1.51

[13] Sanz, M. et al. Periodontitis and cardiovascular diseases: consensus report. *J. Clin. Periodontol.* **47**, 268–288 (2020). 32011025 10.1111/jcpe.13189 PMC7027895

[14] Barr, C., Lopez, M. R. & Rua‐Dobles, A. Periodontal changes by HIV serostatus in a cohort of homosexual and bisexual men. *J. Clin. Periodontol.* **19**, 794–801 (1992). 1452807 10.1111/j.1600-051x.1992.tb02173.x

[15] Bruske, J. S. Periodontoclasia—a working hypothesis. *J. Am. Dent. Assoc. (1922)* **15**, 723–728 (1928).

[16] Slots, J. Microflora in the healthy gingival sulcus in man. *Eur. J. Oral. Sci.* **85**, 247–254 (1977). 10.1111/j.1600-0722.1977.tb00560.x 17152

[17] Socransky, S. S. Microbiology of periodontal disease—present status and future considerations. *J. Periodontol.* **48**, 497–504 (1977). 333085 10.1902/jop.1977.48.9.497

[18] Tanner, A. C. R., Haffer, C., Bratthall, G. T., Visconti, R. A. & Socransky, S. S. A study of the bacteria associated with advancing periodontitis in man. *J. Clin. Periodontol.* **6**, 278–307 (1979). 294457 10.1111/j.1600-051x.1979.tb01931.x

[19] Moore, W. E. et al. Bacteriology of severe periodontitis in young adult humans. *Infect. Immun.* **38**, 1137–1148 (1982). 7152665 10.1128/iai.38.3.1137-1148.1982 PMC347868

[20] Loesche, W. J. The specific plaque hypothesis and the antimicrobial treatment of periodontal disease. *Dent. Update* **19**, 68 (1992). 1291362

[21] Loesche, W. J. Clinical and microbiological aspects of chemotherapeutic agents used according to the specific plaque hypothesis. *J. Dent. Res.* **58**, 2404–2412 (1979). 41862 10.1177/00220345790580120905

[22] Socransky, S. S., Haffajee, A. D., Cugini, M. A., Smith, C. & Kent, R. L. Microbial complexes in subgingival plaque. *J. Clin. Periodontol.* **25**, 134–144 (1998). 9495612 10.1111/j.1600-051x.1998.tb02419.x

[23] Sela, M. N. Role of *treponema denticola* in periodontal diseases. *Crit. Rev. Oral. Biol. Med.* **12**, 399–413 (2001). 12002822 10.1177/10454411010120050301

[24] Sheets, S. M. Gingipain-dependent interactions with the host are important for survival of *Porphyromonas gingivalis*. *Front. Biosci.* **ume**, 3215 (2008). 10.2741/2922 PMC3403687 18508429

[25] Meyer, D. H. & Fives-Taylor, P. M. The role of *Actinobacillus actinomycetemcomitans* in the pathogenesis of periodontal disease. *Trends Microbiol.* **5**, 224–228 (1997). 9211642 10.1016/S0966-842X(97)01055-X

[26] Mysak, J. et al. *Porphyromonas gingivalis*: major periodontopathic pathogen overview. *J. Immunol. Res.* **2014**, 1–8 (2014). 10.1155/2014/476068 PMC3984870 24741603

[27] Jain, S. & Darveau, R. P. Contribution of *Porphyromonas gingivalis* lipopolysaccharide to periodontitis. *Periodontol.* **54**, 53–70 (2010). 10.1111/j.1600-0757.2009.00333.x PMC2943730 20712633

[28] Li, M., Zhang, C., Jin, L., Matsuo, K. & Yang, Y. *Porphyromonas gingivalis* lipopolysaccharide regulates ephrin/Eph signalling in human periodontal ligament fibroblasts. *J. Periodontal Res.* **52**, 913–921 (2017). 28590061 10.1111/jre.12463 PMC5600103

[29] Monasterio, G. et al. Capsular‐defective *Porphyromonas gingivalis* mutant strains induce less alveolar bone resorption than W50 wild‐type strain due to a decreased Th1/Th17 immune response and less osteoclast activity. *J. Periodontol.* **90**, 522–534 (2019). 30397909 10.1002/JPER.18-0079

[30] Hajishengallis, G. & Lamont, R. J. Beyond the red complex and into more complexity: the polymicrobial synergy and dysbiosis (PSD) model of periodontal disease etiology. *Mol. Oral. Microbiol.* **27**, 409–419 (2012). 23134607 10.1111/j.2041-1014.2012.00663.x PMC3653317

[31] Tadjoedin, F. M. et al. The red and orange complex subgingival microbiome of cognitive impairment and cognitively normal elderly with periodontitis. *Geriatrics* **7**, 12 (2022). 35076522 10.3390/geriatrics7010012 PMC8788293

[32] Aleksijević, L. H. et al. *Porphyromonas gingivalis* virulence factors and clinical significance in periodontal disease and coronary artery diseases. *Pathogens* **11**, 1173 (2022). 36297228 10.3390/pathogens11101173 PMC9609396

[33] Marsh, P. D. Are dental diseases examples of ecological catastrophes? *Microbiology* **149**, 279–294 (2003). 12624191 10.1099/mic.0.26082-0

[34] Ryan, R. J. The accuracy of clinical parameters in detecting periodontal disease activity. *J. Am. Dent. Assoc.* **111**, 753–760 (1985). 3905909 10.14219/jada.archive.1985.0195

[35] American academy of periodontology task force report on the update to the 1999 classification of periodontal diseases and conditions. *J. Periodontol.* **86**, 835–838 (2015). 10.1902/jop.2015.157001 26125117

[36] Lang, N. P., Joss, A., Orsanic, T., Gusberti, F. A. & Siegrist, B. E. Bleeding on probing. A predictor for the progression of periodontal disease? *J. Clin. Periodontol.* **13**, 590–596 (1986). 3489010 10.1111/j.1600-051x.1986.tb00852.x

[37] Lang, N. P., Adler, R., Joss, A. & Nyman, S. Absence of bleeding on probing. An indicator of periodontal stability. *J. Clin. Periodontol.* **17**, 714–721 (1990). 2262585 10.1111/j.1600-051x.1990.tb01059.x

[38] Joss, A., Adler, R. & Lang, N. P. Bleeding on probing. A parameter for monitoring periodontal conditions in clinical practice. *J. Clin. Periodontol.* **21**, 402–408 (1994). 8089242 10.1111/j.1600-051x.1994.tb00737.x

[39] Claffey, N., Nylund, K., Kiger, R., Garrett, S. & Egelberg, J. Diagnostic predictability of scores of plaque, bleeding, suppuration and probing depth for probing attachment loss. *J. Clin. Periodontol.* **17**, 108–114 (1990). 2406292 10.1111/j.1600-051x.1990.tb01071.x

[40] Socransky, S. S., Haffajee, A. D., Goodson, J. M. & Lindhe, J. New concepts of destructive periodontal disease. *J. Clin. Periodontol.* **11**, 21–32 (1984). 6582072 10.1111/j.1600-051x.1984.tb01305.x

[41] Teles, R. et al. Patterns of periodontal disease progression based on linear mixed models of clinical attachment loss. *J. Clin. Periodontol.* **45**, 15–25 (2018). 28985450 10.1111/jcpe.12827

[42] Scannapieco, F. A. Monitoring the efficacy of plaque control methods. *Periodontol. 2000* **8**, 24–41 (1995). 9567944 10.1111/j.1600-0757.1995.tb00043.x

[43] Azevedo, C., Henriques, P., Pannuti, C. & Michel-Crosato, E. Selfie dental plaque index: A new tool for dental plaque assessment. *J. Clin. Exp. Dent.* **11**, 926–931 (2022). 10.4317/jced.59908 PMC9701342 36458034

[44] Berezow, A. B. & Darveau, R. P. Microbial shift and periodontitis. *Periodontol. 2000* **55**, 36–47 (2011). 21134227 10.1111/j.1600-0757.2010.00350.x PMC3058494

[45] Mombelli, A. Microbial colonization of the periodontal pocket and its significance for periodontal therapy. *Periodontol. 2000* **76**, 85–96 (2018). 29193304 10.1111/prd.12147

[46] Haque, M., Sartelli, M. & Haque, S. Dental infection and resistance—global health consequences. *Dent. J. (Basel)* **7**, 22 (2019). 30823670 10.3390/dj7010022 PMC6473604

[47] Martyniak, A. et al. Prevention and health benefits of prebiotics, probiotics and postbiotics in acute lymphoblastic leukemia. *Microorganisms* **11**, 1775 (2023). 37512947 10.3390/microorganisms11071775 PMC10384688

[48] Teughels, W., Van Essche, M., Sliepen, I. & Quirynen, M. Probiotics and oral healthcare. *Periodontol. 2000* **48**, 111–147 (2008). 18715361 10.1111/j.1600-0757.2008.00254.x

[49] Yu, Z. et al. The role of potential probiotic strains Lactobacillus reuteri in various intestinal diseases: new roles for an old player. *Front Microbiol* **14**, 1095555 (2023). 36819028 10.3389/fmicb.2023.1095555 PMC9932687

[50] Plaza-Diaz, J., Ruiz-Ojeda, F. J., Gil-Campos, M. & Gil, A. Mechanisms of action of probiotics. *Adv. Nutr.* **10**, S49–S66 (2019). 30721959 10.1093/advances/nmy063 PMC6363529

[51] Ahire, J. J., Jakkamsetty, C., Kashikar, M. S., Lakshmi, S. G. & Madempudi, R. S. In vitro evaluation of probiotic properties of *Lactobacillus plantarum* UBLP40 isolated from traditional indigenous fermented food. *Probiotics Antimicrob. Proteins* **13**, 1413–1424 (2021). 33761096 10.1007/s12602-021-09775-7

[52] Guo, N. & Lv, L. Mechanistic insights into the role of probiotics in modulating immune cells in ulcerative colitis. *Immun. Inflamm. Dis.* **11**, e1045 (2023). 37904683 10.1002/iid3.1045 PMC10571014

[53] Hemarajata, P. & Versalovic, J. Effects of probiotics on gut microbiota: mechanisms of intestinal immunomodulation and neuromodulation. *Ther. Adv. Gastroenterol.* **6**, 39–51 (2013). 10.1177/1756283X12459294 PMC3539293 23320049

[54] Barzegari, A. et al. The battle of probiotics and their derivatives against biofilms. *Infect. Drug Resist* **13**, 659–672 (2020). 32161474 10.2147/IDR.S232982 PMC7049744

[55] Tomás, M. S. J., Claudia Otero, M., Ocaña, V. & Elena Nader-Macías, M. Production of antimicrobial substances by lactic acid bacteria I: determination of hydrogen peroxide. *Public Health Microbiol.* **268**, 337–346 (2023). 10.1385/1-59259-766-1:337 15156044

[56] Wang, Y., Andrukhov, O. & Rausch-Fan, X. Oxidative stress and antioxidant system in periodontitis. *Front. Physiol.* **8**, 910 (2017). 29180965 10.3389/fphys.2017.00910 PMC5693842

[57] Ishikawa, K. H., Bueno, M. R., Kawamoto, D., Simionato, M. R. L. & Mayer, M. P. A. Lactobacilli postbiotics reduce biofilm formation and alter transcription of virulence genes of aggregatibacter actinomycetemcomitans. *Mol. Oral. Microbiol.* **36**, 92–102 (2021). 33372378 10.1111/omi.12330

[58] Didilescu, A. C., Chinthamani, S., Scannapieco, F. A. & Sharma, A. NLRP3 inflammasome activity and periodontal disease pathogenesis–a bidirectional relationship. *Oral. Dis.* **30**, 4069–4077 (2024). 38817019 10.1111/odi.15005 PMC11480888

[59] Zhao, Y. et al. The role of inflammasome NLPR3 in the development and therapy of periodontitis. *Int. J. Med. Sci.* **19**, 1603–1614 (2022). 36185327 10.7150/ijms.74575 PMC9515687

[60] Fazal, I., Shetty, B., Yadalam, U., Khan, S. F. & Nambiar, M. Effectiveness of periodontal intervention on the levels of N-terminal pro-brain natriuretic peptide in chronic periodontitis patients. *J. Circ. Biomark.* **11**, 48–56 (2022). 36381349 10.33393/jcb.2022.2454 PMC9644434

[61] Mazziotta, C., Tognon, M., Martini, F., Torreggiani, E. & Rotondo, J. C. Probiotics mechanism of action on immune cells and beneficial effects on human health. *Cells* **12**, 184 (2023). 36611977 10.3390/cells12010184 PMC9818925

[62] Najafi, S., Sotoodehnejadnematalahi, F., Amiri, M. M., Pourshafie, M. R. & Rohani, M. Prophylactic vs. Therapeutic effect of probiotics on the inflammation mediated by the NF-κB pathway in inflammatory bowel conditions. *Biomedicines* **11**, 1675 (2023). 37371769 10.3390/biomedicines11061675 PMC10296125

[63] Martin‐Cabezas, R., Davideau, J., Tenenbaum, H. & Huck, O. Clinical efficacy of probiotics as an adjunctive therapy to non‐surgical periodontal treatment of chronic periodontitis: a systematic review and meta‐analysis. *J. Clin. Periodontol.* **43**, 520–530 (2016). 26970230 10.1111/jcpe.12545

[64] Olvera-Rosales, L. et al. Impact of the gut microbiota balance on the health–disease relationship: the importance of consuming probiotics and prebiotics. *Foods* **10**, 1261 (2021). 34199351 10.3390/foods10061261 PMC8230287

[65] How, Y. H. & Yeo, S. K. Oral probiotic and its delivery carriers to improve oral health: a review. *Microbiology*. 10.1099/mic.0.001076 (2021). 34351255

[66] Tekce, M. et al. Clinical and microbiological effects of probiotic lozenges in the treatment of chronic periodontitis: a 1 year follow‐up study. *J. Clin. Periodontol.* **42**, 363–372 (2015). 25728888 10.1111/jcpe.12387

[67] Teughels, W. et al. Clinical and microbiological effects of *Lactobacillus reuteri* probiotics in the treatment of chronic periodontitis: a randomized placebo‐controlled study. *J. Clin. Periodontol.* **40**, 1025–1035 (2013). 24164569 10.1111/jcpe.12155 PMC3908359

[68] Laleman, I., Pauwels, M., Quirynen, M. & Teughels, W. A dual‐strain *Lactobacilli reuteri* probiotic improves the treatment of residual pockets: a randomized controlled clinical trial. *J. Clin. Periodontol.* **47**, 43–53 (2020). 31520543 10.1111/jcpe.13198 PMC6973056

[69] Invernici, M. M. et al. Effects of *Bifidobacterium* probiotic on the treatment of chronic periodontitis: a randomized clinical trial. *J. Clin. Periodontol.* **45**, 1198–1210 (2018). 30076613 10.1111/jcpe.12995 PMC6221043

[70] Grusovin, M. G. et al. Clinical efficacy of *Lactobacillus reuteri*-containing lozenges in the supportive therapy of generalized periodontitis stage III and IV, grade C: 1 year results of a double-blind randomized placebo-controlled pilot study. *Clin. Oral. Investig.* **24**, 2015–2024 (2020). 31620939 10.1007/s00784-019-03065-x

[71] Boyeena, L., Koduganti, R. R., Panthula, V. R. & Jammula, S. P. Comparison of efficacy of probiotics versus tetracycline fibers as adjuvants to scaling and root planing. *J. Indian Soc. Periodontol.* **23**, 539–544 (2019). 31849399 10.4103/jisp.jisp_590_18 PMC6906905

[72] Pudgar, P. et al. Probiotic strains of *Lactobacillus brevis* and *Lactobacillus plantarum* as adjunct to non-surgical periodontal therapy: 3 month results of a randomized controlled clinical trial. *Clin. Oral. Investig.* **25**, 1411–1422 (2021). 32666349 10.1007/s00784-020-03449-4

[73] Twetman, S. et al. Short-term effect of chewing gums containing probiotic *Lactobacillus reuteri* on the levels of inflammatory mediators in gingival crevicular fluid. *Acta Odontol. Scand.* **67**, 19–24 (2009). 18985460 10.1080/00016350802516170

[74] Vohra, F. et al. Effectiveness of scaling and root planing with and without adjunct probiotic therapy in the treatment of chronic periodontitis among *shamma* users and non‐users: a randomized controlled trial. *J. Periodontol.* **91**, 1177–1185 (2020). 31985066 10.1002/JPER.19-0464

[75] İnce, G. et al. Clinical and biochemical evaluation of lozenges containing *Lactobacillus reuteri* as an adjunct to non‐surgical periodontal therapy in chronic periodontitis. *J. Periodontol.* **86**, 746–754 (2015). 25741580 10.1902/jop.2015.140612

[76] European Federation of Periodontology. *New Classification of Periodontal and Peri-Implant Diseases*. 10.1002/JPER.18-0157 (2019).

[77] Morales, A. et al. Clinical effects of *Lactobacillus rhamnosus* in non‐surgical treatment of chronic periodontitis: a randomized placebo‐controlled trial with 1 Year follow‐up. *J. Periodontol.* **87**, 944–952 (2016). 26944407 10.1902/jop.2016.150665

[78] Sajedinejad, N. et al. *Lactobacillus salivarius* NK02: a potent probiotic for clinical application in mouthwash. *Probiotics Antimicrob. Proteins* **10**, 485–495 (2018). 28631250 10.1007/s12602-017-9296-4

[79] Penala, S. et al. Efficacy of local use of probiotics as an adjunct to scaling and root planing in chronic periodontitis and halitosis: a randomized controlled trial. *J. Res Pharm. Pr.* **5**, 86 (2016). 10.4103/2279-042X.179568 PMC4843589 27162801

[80] Pelekos, G., Ho, S. N., Acharya, A., Leung, W. K. & McGrath, C. A double‐blind, paralleled‐arm, placebo‐controlled and randomized clinical trial of the effectiveness of probiotics as an adjunct in periodontal care. *J. Clin. Periodontol.* **46**, 1217–1227 (2019). 31479530 10.1111/jcpe.13191

[81] Alshareef, A. et al. Effectiveness of probiotic lozenges in periodontal management of chronic periodontitis patients: clinical and immunological study. *Eur. J. Dent.* **14**, 281–287 (2020). 32438428 10.1055/s-0040-1709924 PMC7274828

[82] Morales, A. et al. Microbiological and clinical effects of probiotics and antibiotics on nonsurgical treatment of chronic periodontitis: a randomized placebo- controlled trial with 9 month follow-up. *J. Appl. Oral. Sci.* **26**, e20170075 (2018). 29364340 10.1590/1678-7757-2017-0075 PMC5777419

[83] Elsadek, M. F., Ahmed, B. M., Alkhawtani, D. M. & Zia Siddiqui, A. A comparative clinical, microbiological and glycemic analysis of photodynamic therapy and *Lactobacillus reuteri* in the treatment of chronic periodontitis in type-2 diabetes mellitus patients. *Photodiagnosis Photodyn. Ther.* **29**, 101629 (2020). 31870899 10.1016/j.pdpdt.2019.101629

[84] Bazyar, H. et al. The impacts of synbiotic supplementation on periodontal indices and biomarkers of oxidative stress in type 2 diabetes mellitus patients with chronic periodontitis under non-surgical periodontal therapy. A double-blind, placebo-controlled trial. *Diabetes Metab. Syndr. Obes.* **13**, 19–29 (2020). 32021348 10.2147/DMSO.S230060 PMC6954633

[85] Chandra, R. V. et al. Effect of a locally delivered probiotic-prebiotic mixture as an adjunct to scaling and root planing in the management of chronic periodontitis. *J. Int Acad. Periodontol.* **18**, 67–75 (2016). 31473711

[86] Ikram, S. et al. Effect of local probiotic (*Lactobacillus reuteri*) vs systemic antibiotic therapy as an adjunct to non‐surgical periodontal treatment in chronic periodontitis. *J. Investig. Clin. Dent.* **10**, e12393 (2019). 30663271 10.1111/jicd.12393

[87] Vivekananda, M. R., Vandana, K. L. & Bhat, K. G. Effect of the probiotic *Lactobacilli reuteri* (Prodentis) in the management of periodontal disease: a preliminary randomized clinical trial. *J. Oral. Microbiol* **2**, 5344 (2010). 10.3402/jom.v2i0.5344 PMC3084569 21523225

[88] Iwasaki, K. et al. Daily intake of heat-killed Lactobacillus plantarum L-137 decreases the probing depth in patients undergoing supportive periodontal therapy. *Oral. Health Prev. Dent.* **14**, 207–14 (2016). 27175447 10.3290/j.ohpd.a36099

[89] Gheisary, Z. et al. The clinical, microbiological, and immunological effects of probiotic supplementation on prevention and treatment of periodontal diseases: a systematic review and meta-analysis. *Nutrients* **14**, 1036 (2022). 35268009 10.3390/nu14051036 PMC8912513

[90] Ausenda, F. et al. Clinical, microbiological and immunological short, medium and long-term effects of different strains of probiotics as an adjunct to non-surgical periodontal therapy in patients with periodontitis. Systematic review with meta-analysis. *Jpn. Dent. Sci. Rev.* **59**, 62–103 (2023). 36915665 10.1016/j.jdsr.2023.02.001 PMC10006838

[91] Li, J., Zhao, G., Zhang, H. & Zhu, F. Probiotic adjuvant treatment in combination with scaling and root planing in chronic periodontitis: a systematic review and meta-analysis. *Beneficial Microbe.* **14**, 95–107 (2023). 10.3920/BM2022.0056 36856123

[92] Sanz, M. et al. Treatment of stage I–III periodontitis—the EFP S3 level clinical practice guideline. *J. Clin. Periodontol.* **47**, 4–60 (2020). 32383274 10.1111/jcpe.13290 PMC7891343

[93] Morales, A. et al. Clinical effects of probiotic or azithromycin as an adjunct to scaling and root planning in the treatment of stage III periodontitis: a pilot randomized controlled clinical trial. *BMC Oral. Health* **21**, 12 (2021). 33413320 10.1186/s12903-020-01276-3 PMC7792194

[94] Jebin, A., Nisha, K. & Padmanabhan, S. Oral microbial shift following 1 month supplementation of probiotic chewable tablets containing Lactobacillus reuteri UBLRu-87 as an adjunct to phase 1 periodontal therapy in chronic periodontitis patients: a randomized controlled clinical trial. *Contemp. Clin. Dent.* **12**, 121 (2021). 34220150 10.4103/ccd.ccd_135_20 PMC8237819

